# Static and Evolving Norovirus Genotypes: Implications for Epidemiology and Immunity

**DOI:** 10.1371/journal.ppat.1006136

**Published:** 2017-01-19

**Authors:** Gabriel I. Parra, R. Burke Squires, Consolee K. Karangwa, Jordan A. Johnson, Cara J. Lepore, Stanislav V. Sosnovtsev, Kim Y. Green

**Affiliations:** 1 Caliciviruses Section, Laboratory of Infectious Diseases, National Institute of Allergy and Infectious Diseases, National Institutes of Health, Bethesda, MD, United States of America; 2 Bioinformatics and Computational Biosciences Branch, National Institute of Allergy and Infectious Diseases, National Institutes of Health, Bethesda, MD, United States of America; 3 Division of Viral Products, Food and Drug Administration, Silver Spring, MD, United States of America; Tulane University, UNITED STATES

## Abstract

Noroviruses are major pathogens associated with acute gastroenteritis worldwide. Their RNA genomes are diverse, with two major genogroups (GI and GII) comprised of at least 28 genotypes associated with human disease. To elucidate mechanisms underlying norovirus diversity and evolution, we used a large-scale genomics approach to analyze human norovirus sequences. Comparison of over 2000 nearly full-length ORF2 sequences representing most of the known GI and GII genotypes infecting humans showed a limited number (≤5) of distinct intra-genotypic variants within each genotype, with the exception of GII.4. The non-GII.4 genotypes were comprised of one or more intra-genotypic variants, with each variant containing strains that differed by only a few residues over several decades (remaining “static”) and that have co-circulated with no clear epidemiologic pattern. In contrast, the GII.4 genotype presented the largest number of variants (>10) that have evolved over time with a clear pattern of periodic variant replacement. To expand our understanding of these two patterns of diversification (“static” versus “evolving”), we analyzed using NGS the nearly full-length norovirus genome in healthy individuals infected with GII.4, GII.6 or GII.17 viruses in different outbreak settings. The GII.4 viruses accumulated mutations rapidly within and between hosts, while the GII.6 and GII.17 viruses remained relatively stable, consistent with their diversification patterns. Further analysis of genetic relationships and natural history patterns identified groupings of certain genotypes into larger related clusters designated here as “immunotypes”. We propose that “immunotypes” and their evolutionary patterns influence the prevalence of a particular norovirus genotype in the human population.

## Author Summary

Efforts are underway to develop vaccines against norovirus, a leading cause of acute gastroenteritis. The purpose of our study was to understand how norovirus strains within different genotypes evolve and adapt as they are transmitted in the human population. Using large-scale genomics and computational tools developed in our laboratory, we identified two strikingly different evolutionary patterns among norovirus genotypes: “static” and “evolving.” We mined large datasets from infection and outbreak studies in context of these evolutionary patterns and propose a new model for antigenic clustering of norovirus genotypes that could simplify vaccine design.

## Introduction

RNA viruses evolve quickly, with mutation rates that vary between 10^−3^–10^−4^ nucleotide (nt) substitutions/year; up to 1000 times higher when compared with most DNA viruses [1]. This high mutation rate is attributed largely to the inability of their RNA polymerases to correct errors introduced during replication. In addition to nt substitutions, RNA viruses generate diversity by recombination or rearrangements of their genome [2–4]. This diversity is considered required for the virus to (i) escape the immune surveillance of the host [5–7], (ii) reach different organs of the host and/or change tropism, and (iii) induce the pathological effects that could lead to efficient transmission to a new host [8]. Although extreme diversity might be advantageous for a virus to exploit different niches, viruses can also retain their phenotypic characteristics for decades (e.g., dengue virus and respiratory syncytial virus) [5, 9].

Noroviruses are a major cause of acute gastroenteritis worldwide, mainly associated with outbreaks occurring in closed settings, such as hospitals, nursing homes, schools, cruise ships, and military facilities. In countries where rotavirus vaccination has been successfully introduced, norovirus has become the major cause of gastroenteritis in children [10–13]. It is estimated that noroviruses cause between 70,000 to 200,000 deaths per year worldwide, with the majority in children from developing countries [14, 15]. Although symptoms characteristically resolve within 72 hours in healthy immunocompetent individuals, norovirus genomic RNA can be detected in stool for up to 2 months [16–19], and clearance of virus seems to be associated with a specific immune response in the mucosa [18]. In immunocompromised patients, symptoms and virus can persist over years, complicating the management of their underlying disease [20].

Noroviruses are considered fast-evolving viruses [21–24], and present an extensive diversity that is driven by acquisition of point mutations and recombination. The genome consists of a single-stranded positive-sense RNA molecule of ~7.5kb that is organized into three open reading frames (ORFs). ORF1 encodes a polyprotein that is co-translationally cleaved into six proteins required for replication, while ORF2 encodes the major capsid protein (VP1), and ORF3, a minor capsid protein (VP2) [25]. Expression of recombinant VP1 yields virus-like particles (VLPs) that mimic the native virion, which have been important research tools in the absence of tractable cell culture systems and animal models [26–30]. Based on sequence differences of the VP1 protein, noroviruses have been classified into seven genogroups (GI-GVII) and over 30 genotypes [25, 31]. Despite this extensive diversity, a single genotype (GII.4) has been shown to be the most prevalent in humans worldwide [31–33]. Since the mid-1990s, six global epidemics have been documented and each has been associated with the emergence of a new GII.4 variant. The first was characterized by an increased number of norovirus outbreaks worldwide, and associated with the US95_1996 virus. The second epidemic started in 2002 and coincided with the replacement of the US95_1996 virus by the Farmington_Hills_2002 virus. The third epidemic was caused by the Hunter_2004 virus, which was rapidly replaced by two new pandemic strains, namely Den_Haag_2006b and Yerseke_2006a. During 2009, a new strain emerged (New_Orleans_2009) that co-circulated with the 2006 variants for almost three years until the current predominant variant emerged in 2012 (Sydney_2012) [25, 31, 33]. Interestingly, this epidemiological pattern has not been reported for any other norovirus genotype, until recently. A novel GII.17 variant has emerged, potentially displacing an older GII.17, causing large outbreaks in different countries from Asia [22, 34–36]. Although GII.4 is overall the most prevalent genotype in the human population, multiple norovirus genotypes co-circulate in children with low to high incidence. Genotypes GII.3, GII.6 and GII.2 (in addition to GII.4) have consistently been linked to infection in children under 5 years of age [17, 21, 37, 38].

Initial challenge studies in human volunteers suggested a lack of protective responses between strains from the two major genogroups (GI and GII), as cross-challenge between Norwalk virus (the GI.1 prototype strain) and Hawaii virus (the GII.1 prototype strain) did not induce protection. In addition, duration of immunity might be short (less than 6 months), as individuals re-challenged with the same virus became ill during the second exposure [39]. It has been noted that the high titer of challenge virus administered in the early volunteer studies might not reflect that during natural exposure [40, 41] and recent studies have focused on the natural history of noroviruses. Based on epidemiological data, Simmons et al. modeled that norovirus genotype-specific immunity could last up to 9 years [42], which would enhance the duration of vaccine-induced immunity. The diversity of genotypes has also been addressed. Children can be re-infected multiple times during the first 5 years of life [17, 18, 38]_,_ with the majority of re-infections occurring with different norovirus genotypes. These data suggest that genotypes may represent distinct serotypes, which would complicate vaccine design.

In this study, we integrated large-scale genomics analysis with natural history data to investigate mechanisms involved in the diversification and evolution of norovirus genotypes and their variants. Most norovirus intra-genotypic variants displayed a striking genetic stability over long periods of time, with GII.4 as the notable exception. We detected patterns of re-infection and susceptibility consistent with genetic and antigenic clustering of certain genotypes and propose that these relationships may be relevant in the design of norovirus vaccines.

## Results

### Intra-genotypic diversity

**Fig 1:**
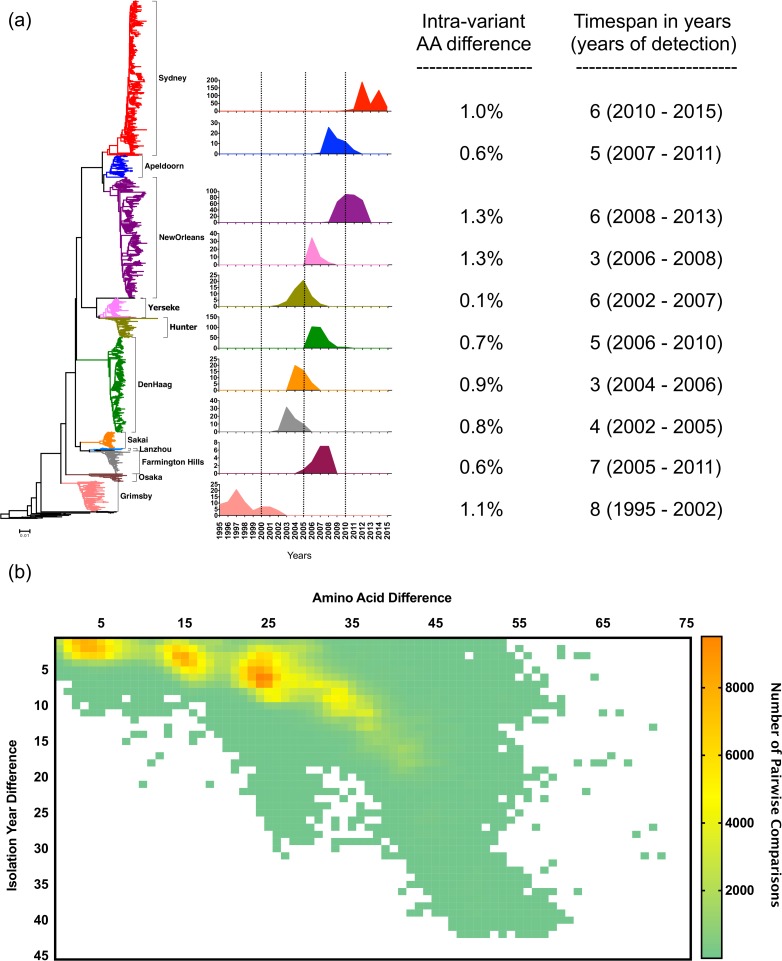
Diversity and evolutionary pattern of GII.4 noroviruses. (a) Phylogenetic tree showing the presence of multiple variants within the GII.4 genotype. Each variant is identified by a different color and name. Sequences from each variant were collected from a period of 3–8 years (mean: 5.3 years) until replaced by a novel variant. Tree was constructed using nt sequences encoding the VP1 and Kimura 2-parameter and Neighbor-Joining method as implemented in MEGA v6. Plots indicate the number of sequences collected each year from ten major GII.4 variants. (b) Diversity plot showing the accumulation of aa mutations over time in the VP1 from GII.4 viruses. Note that a higher number of strains accumulate at ~5% aa difference (25/539) and 5 years of detection difference, which correlates with the timespan where a novel variant displaces the old one. Heat map represents the number of pairwise comparisons, red being the highest and green the lowest number of pairwise comparisons.

**Fig 2:**
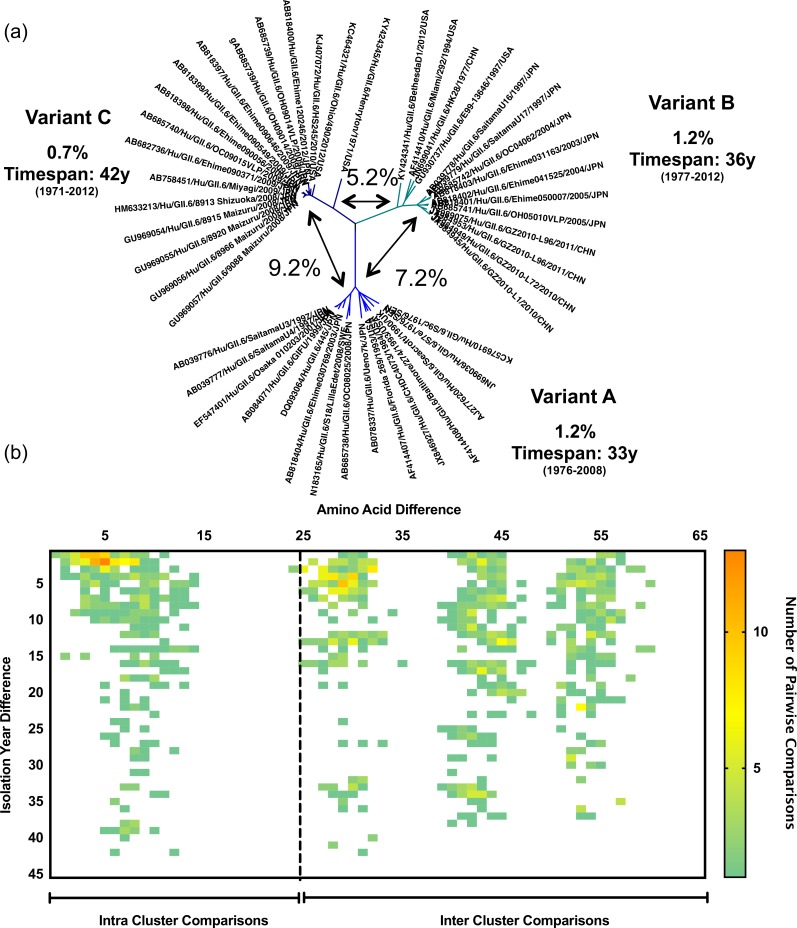
Diversity and evolutionary pattern of GII.6 viruses. (a) Phylogenetic tree showing the presence of three variants (A-C) within the GII.6 genotype. Each variant branch is identified by different shades of blue. The timespan of isolation of the oldest and newest strain and the percent amino acid difference within each variant is shown. Arrows between branches represent the percent amino acid difference between variants. Tree was constructed using nt sequences encoding the VP1 and Kimura-2 parameter and Neighbor-Joining method as implemented in MEGA v6. (b) Diversity plot showing the presence of discrete values representing differences within and between the three GII.6 variants. The GII.6 genotype has strains that differ by only a few aa (<10) but identified over 40 years apart, or strains can differ by almost 57 aa and detected in the same year. Heat map represents the number of pairwise comparisons, red being the highest and green the lowest number of pairwise comparisons. Similar data were generated for 14 other norovirus genotypes.

To investigate the diversity and evolutionary differences of the distinct norovirus genotypes, more than 2000 sequences of the gene (ORF2) encoding the VP1 were retrieved from GenBank, with 101 and 1909 genes from GI strains and GII strains, respectively (Table 1). Individual sequences were vetted with an online norovirus typing tool that follows a widely-used universal classification and nomenclature system for norovirus genotypes [25], and an effort was made to verify the date of occurrence with the supporting documentation. Genotypes with 10 or more complete (or nearly complete) ORF2 sequences were selected for further phylogenetic analysis at both the nt and amino acid (aa) level, and included 16 out of the 31 current GI and GII genotypes (Table 1). We defined an intra-genotypic variant as a group of strains (≥ 2) that clustered together in the phylogenetic tree and that showed <5% difference in their nt or aa sequences, but ≥5% difference compared to other strains. Most genotypes segregated into 1 to 5 phylogenetic variants when nt sequences were analyzed (Table 1), with the exception of genotype GII.4 that displayed at least 10 different variants (Table 1 and Fig 1A). The number of variants was lower in seven genotypes (GI.1, GI.4, GI.6, GII.2, GII.12, GII.13 and GII.14) when aa sequences were used for tree reconstruction and distance analyses (Table 1). To link the different intra-genotypic variants to phenotypic characteristics in the VP1, we focused on the analysis of aa sequences. Interestingly, non-GII.4 genotypes presented variants with strains that have been detected many years apart (mean: 24.9 years, standard deviation: 12.97 years) while having only a few differences in their aa sequence (Table 1). An example is the GII.6 genotype, with each of the three variants (A-C) containing strains that differed by only a few aa residues (≤1.2%) but that were detected up to 41 years apart (Fig 2A). In contrast, the GII.4 genotype was comprised of variants that were present in the human population from 3 to 8 years (mean: 5.3 years, Fig 1A).

**Table 1:** Summary of the sequence analyses conducted for each norovirus genotype.

Genotype	Main Host		Number of Sequences Retrieved	Number of Variants^1^		dN/dS		Mean Evolutionary Rate (nucleotide substitutions/site/year)	AA-based Evolutionary Pattern^2^	Timespan (years) between Distinct Variants within Each Genotype
				NT-based Tree	AA-based Tree	VP1	P2 domain			
**GI.1**		**Human**	**13**	**2**	**1**	**0.03**	**0.04**	**1.16E-03**	**Static**	**39**
**GI.2**		**Human**	**8**	**1**	**1**	**-**	**-**	**-**	**-**	**-**
**GI.3**		**Human**	**27**	**5**	**5**	**0.08**	**0.17**	**2.69E-03**	**Static**	**1/35/31/16/14**
**GI.4**		**Human**	**15**	**5**	**1**	**0.04**	**0.10**	**1.04E-03**	**Static**	**18**
**GI.5**		**Human**	**7**	**2^3^**	**2^3^**	**-**	**-**	**-**	**-**	**-**
**GI.6**		**Human**	**12**	**3**	**2**	**0.05**	**0.07**	**7.05E-04**	**Static**	**17/16**
**GI.7**		**Human**	**9**	**4^3^**	**3^3^**	**-**	**-**	**-**	**-**	**-**
**GI.8**		**Human**	**5**	**1**	**1**	**-**	**-**	**-**	**-**	**-**
**GI.9**		**Human**	**5**	**1**	**1**	**-**	**-**	**-**	**-**	**-**
**GII.1**		**Human**	**17**	**2^3^**	**2**	**0.06**	**0.09**	**2.45E-03**	**Static**	**41/20**
**GII.2**		**Human**	**101**	**4**	**2**	**0.04**	**0.07**	**2.99E-03**	**Static**	**40/0**
**GII.3**		**Human**	**118**	**1**	**1**	**0.07**	**0.11**	**3.23E-03**	**Static**	**-**
**GII.4**		**Human**	**1343**	**11**	**11**	**0.12**	**0.25**	**5.40E-03**	**Evolving**	**5.3 (3–8)^4^**
**GII.5**		**Human**	**18**	**1**	**1**	**0.03**	**0.04**	**1.04E-03**	**Static**	**34**
**GII.6**		**Human**	**48**	**3**	**3**	**0.06**	**0.06**	**1.43E-03**	**Static**	**32/35/41**
**GII.7**		**Human**	**13**	**1**	**1**	**0.05**	**0.06**	**1.66E-03**	**Static**	**33**
**GII.8**		**Human**	**2**	**1**	**1**	**-**	**-**	**-**	**-**	**-**
**GII.9**		**Human**	**3**	**1**	**1**	**-**	**-**	**-**	**-**	**-**
**GII.10**		**Human**	**4**	**1**	**1**	**-**	**-**	**-**	**-**	**-**
**GII.11**		**Porcine**	**9**	**1**	**1**	**-**	**-**	**-**	**-**	**-**
**GII.12**		**Human**	**26**	**3^3^**	**1**	**0.04**	**0.06**	**2.17E-03**	**Static^5^**	**20**
**GII.13**		**Human**	**11**	**2**	**1**	**0.07**	**0.15**	**1.71E-03**	**Static^5^**	**29**
**GII.14**		**Human**	**18**	**2^3^**	**1**	**0.08**	**0.11**	**2.23E-04**	**Static^6^**	**35**
**GII.15**		**Human**	**4**	**1**	**1**	**-**	**-**	**-**	**-**	**-**
**GII.16**		**Human**	**12**	**1**	**1**	**0.07**	**0.07**	**6.10E-04**	**Static**	**9**
**GII.17**		**Human**	**144**	**4**	**4**	**0.11**	**0.26**	**1.07E-03**	**Static^7^**	**37/4/1/1**
**GII.18**		**Porcine**	**3**	**1**	**1**	**-**	**-**	**-**	**-**	**-**
**GII.19**		**Porcine**	**2**	**1**	**1**	**-**	**-**	**-**	**-**	**-**
**GII.20**		**Human**	**5**	**2**	**2**	**-**	**-**	**-**	**-**	**-**
**GII.21**		**Human**	**6**	**2**	**2**	**-**	**-**	**-**	**-**	**-**
**GII.22**		**Human**	**2**	**1**	**1**	**-**	**-**	**-**	**-**	**-**

^1^ A variant is defined as a group of strains (≥ 2) that cluster together and have <5% differences in their sequences.

^2^ Evolutionary pattern was determined only for genotypes presenting ≥ 10 sequences.

^3^ Variant including strains with >5% differences in their sequences.

^4^ Average (range) of years for any given GII.4 variant.

^5^ Genotype presents accumulation of mutations over time without reaching cut-off values (≥5%) for variant designation.

^6^ AA-based tree presents 2 phylogenetic groups but they do not reach cut-off values (≥5%) for variant designation.

^7^ Variant A strains with over 30 years differences and minimal (<19 aa) differences in the capsid protein.

We developed an algorithm to illustrate visually the relationship between amino acid diversity and time among strains from a given genotype. The algorithm generated a heat map in which each square represents the number of strains with such aa difference in their VP1 and plotted against the timespan of detection. Analysis of 16 genotypes with sufficient data in GenBank revealed two distinct patterns of variant evolution; one in which the number of aa differences accumulated continually over time (Fig 1B), and one in which the number of aa differences remained relatively constant over time, regardless of the timespan between strains (Fig 2B). The first pattern (Fig 1B) was related to a constantly changing “evolving genotype” represented by the GII.4, while the second pattern (Fig 2B) was a highly conserved or “static genotype” represented by the 15 other genotypes with sufficient data for analysis (Table 1). The static genotypes resolved readily into distinct intra-genotypic variants, with one exception. The diversity plot of the GII.12 genotype strains displayed a subtle accumulation of differences over a time period of approximately 20 years; however, those differences were not associated with different intra-genotypic variants (S1 Fig). A larger number of sequences over a longer period will be helpful for defining variant diversity in GII.12 and other static genotypes.

During 2014–2015, a sharp increase in the number of gastroenteritis outbreaks was reported in Asia [22, 34, 43] that were associated with the emergence of a new variant of the genotype GII.17, i.e. variant C or Kawasaki_2014 [35, 43]. Our phylogenetic analysis of this genotype revealed four distinct variants, with one of the variants having strains that spanned over 37 years with a low level of sequence diversity consistent with a static pattern (S2 Fig). In contrast, the emerging strains showed multiple substitutions at the nt and aa sequence level [22, 35][44], which led to diversification into two separate phylogenetic variants (C and D). These differences appeared to accumulate over time, with predominant strains that circulated during the 2014–2015 season (variant D) differing by 5.4±1.1% from those in the 2013–2014 season (variant C) in their aa sequence.

To gain insight into the evolutionary differences noted at the aa level, the nt rate of evolution and nonsynonymous substitutions (dN)/synonymous substitutions (dS) ratio were calculated for each of the genotypes included in this analysis. The nt rates of evolution were similar among all the norovirus genotypes (range: 5.40x10^-3^–2.23x10^-4^ nt substitutions/site/year). However, differences were seen in the dN/dS ratios, with genotypes GII.4 and GII.17 presenting slightly higher values than any other norovirus genotype (Table 1). Note that in five different genotypes (GI.3, GI.4, GII.4, GII.13, and GII.17), the dN/dS ratio in the P2 encoding region was at least two times higher than the complete ORF2 (encoding VP1) dN/dS ratio. The dN/dS ratio has been used to inform the evolutionary pressures on a gene: a dN/dS > 1 (higher number of non-synonymous mutations) indicates positive evolution as the phenotype is changing due to pressures of the environment (e.g. immune responses), while a dN/dS < 1 indicates a purifying selection (also known as negative selection), where the new phenotype is mostly deleterious and eliminated from the population [45–47]. Despite the small differences in the dN/dS ratios, purifying selection (dN/dS < 1) is strongly acting at the VP1 protein level in all norovirus genotypes, including GII.4.

### Intra- and inter-host evolution of norovirus genotypes

**Fig 3:**
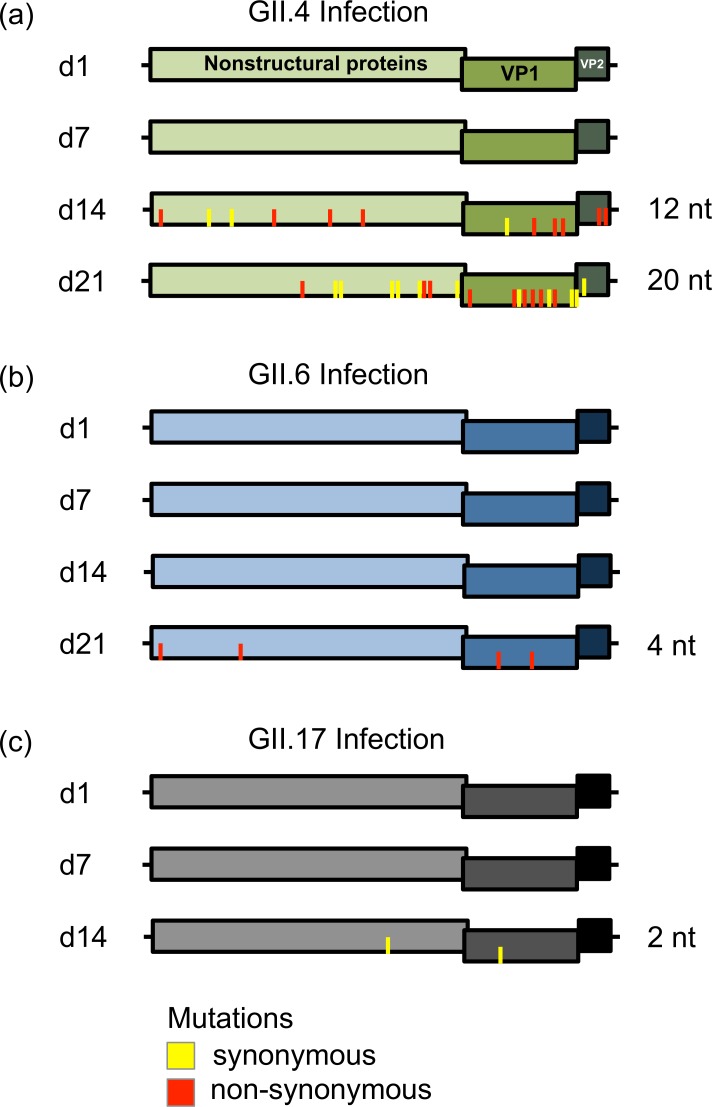
Intra-host diversity of noroviruses in an immunocompetent individual with three sequential norovirus infections. Summary of NGS data from consecutive samples collected from an individual who shed (a) GII.4 (Sydney variant), (b) GII.6 (B variant), or (c) GII.17 (D variant) norovirus for 2–3 weeks (Days 1, 7, 14, and 21). All consensus sequences were identical to the reference (Day 1); however, mutations ranging from 5 to 50% of the total reads (indicated with yellow bars, synonymous, and red bars, non-synonymous) arose at later time points for each virus. GII.4 viruses are indicated in green, GII.6 in blue, and GII.17 in grey.

We next examined whether the different patterns of diversification observed in ORF2 extended to other regions of the genome. We analyzed by NGS full-length norovirus genomes from immunocompetent individuals infected in different settings. The first set of samples was from a child who was consecutively infected with three different genotypes (GII.4, GII.6 and GII.17) over a 3-year period [18, 35]. Although in each episode the child resolved the symptoms within ~ 72 hours, viral RNA was detected in stools for weeks after onset of symptoms. The full-length genome sequences were compared within the first 3 weeks for GII.4 and GII.6 viruses and the first 2 weeks for GII.17 viruses. A total of up to 78,000 reads/site (mean: 14688, standard deviation: 5675) were obtained for each sample by NGS (S1 Table). All consensus sequences were identical to the reference (day 1 [d1]); however, mutations ranging from 5 to 50% of the total reads (S1 Table and Fig 3) were found at later time points for each virus. In GII.4 viruses, 12 nt mutations arose in the subpopulations of the sample collected at d14, with nine of them being non-synonymous mutations. Three aa mutations were located in the P domain, the capsid surface-exposed region of the VP1 protein, with two present in antigenic sites A (E368A) and E (S412N) [48] (S3 Fig). By d21, the GII.4 virus had acquired 20 nt mutations in its subpopulations, with most of them (19/20) new mutations as compared to the d14 sequence. Nine out of the 20 nt mutations changed the aa sequence, with 4 of them mapping to the P domain, near or at antigenic sites (S3 Fig). In contrast, GII.6 and GII.17 viruses presented only four and two substitutions in their subpopulations at d21 and d14, respectively, with no evidence of the accumulation of mutations over time (S2 Table).

**Fig 4:**
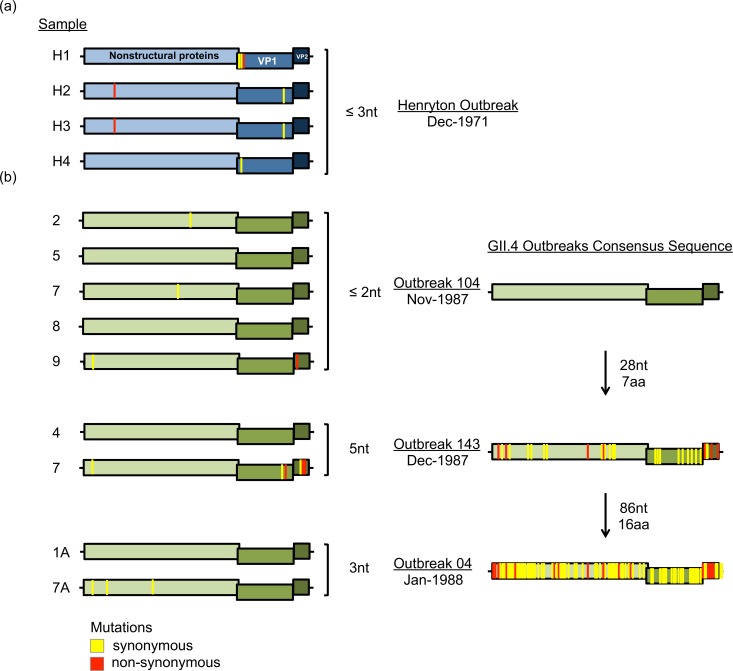
Inter-host diversity of noroviruses in immunocompetent individuals. Summary of NGS data from multiple patient samples collected during discrete outbreaks. Genomes analyzed from the GII.6 outbreak were colored in blue (a), and those from the GII.4 outbreaks in green (b). All viral sequences were nearly identical (≤ 5 nt differences) within each outbreak; however, GII.4 viruses accumulated over 80 nt mutations within a single season (3 months). Red (non-synonymous) and yellow (synonymous) bars indicate substitutions compared with the consensus sequence of the outbreak strain. Note that despite the occurrence of over 80 nt mutations throughout the genome of GII.4 strains within a single season, all mutations that mapped into the VP1 were synonymous.

Although a large amount of norovirus is shed in stool, the infectious dose in natural transmission is likely low [41]. Thus, during inter-host transmission events noroviruses may undergo an initial reduction in the number of replicating viruses, creating a bottleneck effect. To compare inter-host evolution in individuals involved in outbreaks, we analyzed samples from outbreaks that occurred in the state of Maryland where the causative agents were identified as GII.6 (a hospital outbreak in 1971) [49] or GII.4 noroviruses (nursing home outbreaks in the 1987–1988 winter season) [32]. The samples and their dates of collection are indicated in Fig 4. Comparison of the NGS sequences with the outbreak consensus sequence revealed only a few substitutions, ≤ 5 nt and ≤ 2 aa, among samples from the same outbreak (Fig 4A and Fig 4B). However, when the consensus sequences from the GII.4 outbreaks were compared, a progressive accumulation of mutations (up to 86 nt and 16 aa) were detected in a period of three months, with no aa substitutions detected in the ORF2 (Fig 4B). To confirm these observations, we compared 151 genomes from GII.4 viruses (variant Den Haag) detected during three epidemic seasons in Japan [50, 51], and showed the accumulation of nt and aa substitutions over time (S4 Fig). In both sets of samples, the ORF2 (encoding VP1) acquired fewer amino acid substitutions as compared with ORF1 and ORF3, and thus maintained the VP1 phenotype for the GII.4 variant circulating in that given season. The data from full-length genome analyses were consistent with those from analyses of ORF2 in the GenBank database: different patterns of evolution exist among the norovirus genotypes in an acute outbreak setting.

### Norovirus re-infection and genotype clustering

**Fig 5:**
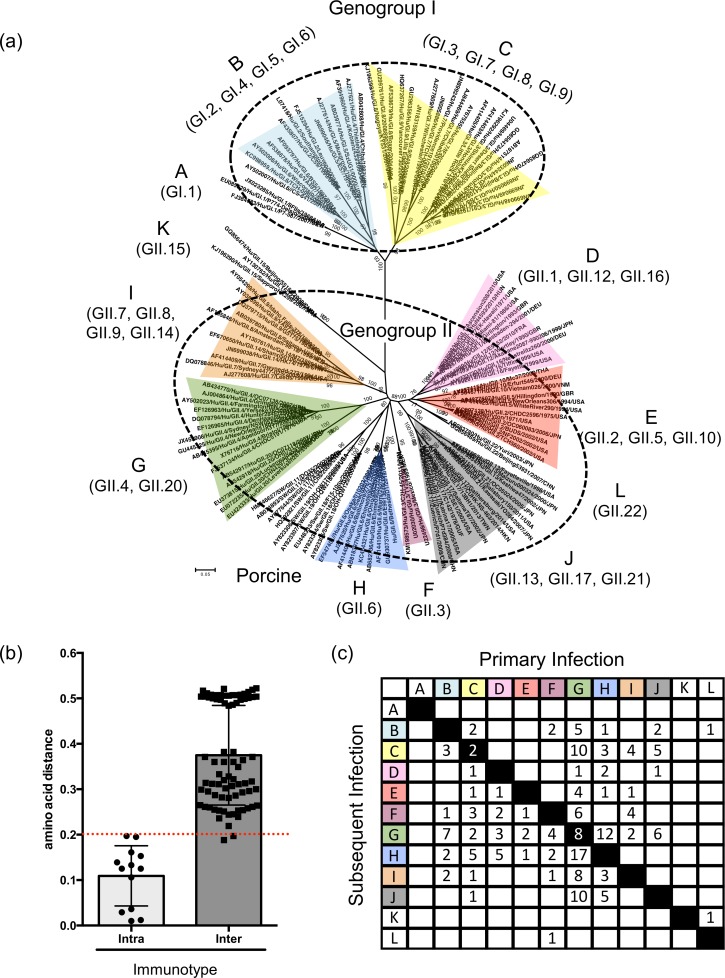
Clustering of human norovirus genotypes into “immunotypes”. (a) Phylogenetic tree showing the presence of 7 clusters containing two or more genotypes and 5 comprised of a single genotype, which define the 12 larger order clusters designated as immunotypes (A-L) for this study. The phylogenetic tree was constructed using three representative strains from each variant described for each genotype except for GII.4, where only one strain from each of the variants was used. The evolutionary distances of the amino acid sequences were computed using the Poisson correction method and the tree inferred using the Neighbor-Joining method and bootstrap test (1000 replicates). (b) Amino acid (aa) differences within (intra-immunotype) and between (inter-immunotype) the proposed immunotypes. The bars indicate the average of the aa differences and standard error. The red dotted line indicates the cut-off value (≥20%) of aa differences between groups. (c) Matrix showing the frequency of re-infection and the genotypes detected. Data was obtained from studies that followed the natural history of norovirus infection [17, 18, 22, 38, 52–54]. Each genotype is grouped within their respective immunotype. Re-infection with strains from the same immunotype are indicated by black cells. Note that the eight cases of re-infection within the immunotype G represent re-infections with different GII.4 variants.

To reconcile our observations on the different mechanisms of diversification and data on re-infection and epidemiology of noroviruses, we investigated whether additional relationships might exist among the genotypes from the two major genogroups. Our phylogenetic tree constructed with representative strains from each genotype (strains from each lineage described here were included) showed clustering among certain genotypes (e.g. GI.3, GI.7, GI.8 and GI.9), while others appeared as single genotypes (e.g. GI.1, GII.3, GII.6; Fig 5A, S3 Table). The genotypic clustering was reproducible with a second phylogenetic methodology (S5 Fig). We designated each of the separate branches as groups A-L (Fig 5A), and the deduced aa sequences showed an approximate cut-off value of ≥20% aa differences between groups (Fig 5B). In a review of data from research groups that have documented norovirus re-infections and determined the genotype for each infection [17, 18, 38, 52–54], we observed that the pattern of re-infection might be consistent with the new grouping system as a predictor of antigenically-distinct strains. To test the hypothesis that these groups, provisionally designated here as “immunotypes,” might play a role in norovirus immunity, we developed a matrix that recorded the data from each of the consecutive re-infection cases with documented norovirus genotyping [17, 18, 22, 38, 52–54]. For example, a child consecutively infected with a GII.4 (“immunotype” G), GII.6 (“immunotype” H), and GII.17 (“immunotype” J) norovirus would count as one individual for the cell of the matrix that compares immunotype G and H, and as one individual for the cell that compares immunotype H and J. A matrix was constructed using re-infection data available from 116 children and 2 adults (Fig 5C, S6 Fig). Overall, the majority of re-infections occurred with viruses from different immunotypes, with re-infection rare from strains within an immunotype. A notable exception was immunotype G, which is comprised of genotypes GII.4 and GII.20. Re-infection of eight children (as shown by the 8 individuals in the black cell) was documented to have occurred with different variants of GII.4 viruses [17, 38].

## Discussion

Viruses are genetically and structurally diverse. Depending on their genome and/or replication strategies, viruses can present different rates of evolution (range: 10^−2^–10^−9^ nt substitutions/site/year) [3, 47, 55]. As with many other RNA viruses, noroviruses have been regarded as rapidly evolving viruses [22, 48, 56]. The overall rate of evolution for the norovirus genotypes included in this study ranged from 5.40x10^-3^–2.23x10^-4^ nt substitutions/site/year for the VP1 encoding region, which were similar to those described previously for norovirus GII.4, GII.3, GII.6, and GI.1-GI.6 [21, 57–61], and within the range for positive-strand RNA viruses [3]. Despite this high nt mutation rate, the number of non-synonymous substitutions were on average ~18 times lower than the synonymous substitution (dN/dS average: 0.06), suggesting that purifying selection (dN/dS <1) acts strongly in the VP1 protein. Similar observations have been made for other RNA viruses, where the rate of evolution reached up to 10^−2^ nt substitutions/site/year (depending on the region of the genome used for analyses) but was mostly dominated by high synonymous substitution rates [46, 55, 62]. In noroviruses, positive selection has been reported for certain codons of the VP1 for GII.4, GII.3, GII.6 and GII.17 viruses [21, 22, 57, 58, 63], and codon changes in the antigenic sites of GII.4 viruses (which are located in loops of the P2 domain) have correlated with the emergence of new variants [24, 27, 48]. Taken together, our findings suggest that the capsid protein of all noroviruses evolve with strong structural constraints, with only a limited number of codons that can evolve and, perhaps confer adaptive advantages to infect human hosts.

Epidemiological studies coupled with sequence data from field isolates have indicated that the most predominant norovirus genotype, GII.4, is evolving similarly to influenza H3N2 viruses; i.e. with a temporal replacement of predominant variants that is driven by the immune response of the host [27, 48, 64]. By exploring the intra-genotypic diversity from representative human norovirus genotypes we verified that GII.4 noroviruses produce the largest number of intra-genotypic variants, and that these variants last (on average) ~5 years in the human population. In contrast, non-GII.4 noroviruses sustain a low number of intra-genotypic variants with a limited number of aa differences among strains within that given variant; even if decades apart in occurrence. Interestingly, different variants from a given genotype can often be co-circulating within the same year and geographical location causing gastroenteritis [37, 44, 65, 66]. The GII.4 viruses, and to a lesser extent one variant of the GII.17 viruses, acquired aa substitutions over time that created phenotypically different variants. In contrast, all other genotypes retained similar sequences within variants that might have arisen early in the origin of that genotype and that persisted over time. This led us to discriminate two different patterns of evolution in norovirus: evolving and static. Evolving viruses continually accumulate mutations in their genome over time, and static viruses do not.

The concept of evolving versus static norovirus genotypes may be helpful in understanding the spread of pandemic strains. The recent emergence of GII.17 viruses resulted in the rapid replacement of one variant (variant C) with another (variant D) [22, 44]. This pattern of very rapid replacement, occurring within two consecutive seasons, in the emerging GII.17 viruses is notably different from that of GII.4 viruses, in which each emerging GII.4 variant is replaced every 3 to 8 years. Thus, since the GII.17 genotype presents other variants shown to be “static,” the recent global spread of the GII.17 genotype might be the moment when a new genotypic variant (variant C) emerged and is quickly adapting to reach maximum fitness in the human host (variant D) to become static. Since the emergence of this GII.17 strain has only recently occurred and most of the available GII.17 sequences (136/143) correspond to these two variants, more information on pre-2013 strains and the future epidemiological behavior of the GII.17 strains will be helpful in establishing the evolutionary pattern of this genotype. Because recombination has been suggested to play an important role on the emergence of many GII.4 variants [67], and the emerging GII.17 strains presented a novel polymerase (encoded by ORF1) [22, 34, 35, 44], further studies should be conducted on the role of recombination in norovirus VP1 diversification into variants.

To determine the role of intra-host evolution at the genomic level, we developed a method to generate and analyze full-length norovirus genomes with NGS technologies and bioinformatics. The strategy of amplification was similar to that published by Eden et al. [67] for GII.4 viruses, and our method was robust for a number of GII noroviruses (GII.1, GII.2, GII.3, GII.4, GII.6, GII.12, and GII.17), and from samples stored for over 40 years [35]. Several groups have explored the intra-host diversity of noroviruses by NGS using partial regions of the genome [23, 68]; however, our approach extended these findings by allowing high-resolution analysis at every nt position in the coding sequence of the genome. We first examined the intra-host evolution of GII.4, GII.6 and GII.17 noroviruses within a single patient, and observed that only the GII.4 viruses presented a gradual increase in the number of mutations, which in some cases resulted in aa substitutions in areas regarded as important antigenic sites. The limited intra-host diversity found during the shedding phase of an infection in immunocompetent individuals contrasts with the vast diversity of viruses found in immunocompromised patients [68]. Due to the diversity found in immunocompromised patients and prolonged shedding, it was suggested that they might be a source of new GII.4 variants to the human population [69]. Noroviruses are highly transmissible; however, there is little evidence that norovirus can be efficiently transmitted during the chronic phase of the infection [19]. A more likely source for new GII.4 variants might be immunocompetent individuals, where we show that mutations can arise during inter-host transmission events, and accumulate during the intra-variant period. Although noroviruses belonging to the “static” genotypes can also accumulate mutations during inter-host transmission events, those mutations would likely be eliminated from the viral population by purifying selection. Viruses that better tolerate the introduction of mutations are regarded as genetically robust, and this robustness has been shown to be beneficial for virus survival and prevalence [70]. Overall our data suggest that GII.4 noroviruses are genetically robust. In contrast, noroviruses with “static” genotypes may be genetically fragile, which limits their antigenic diversity and prevalence.

**Fig 6:**
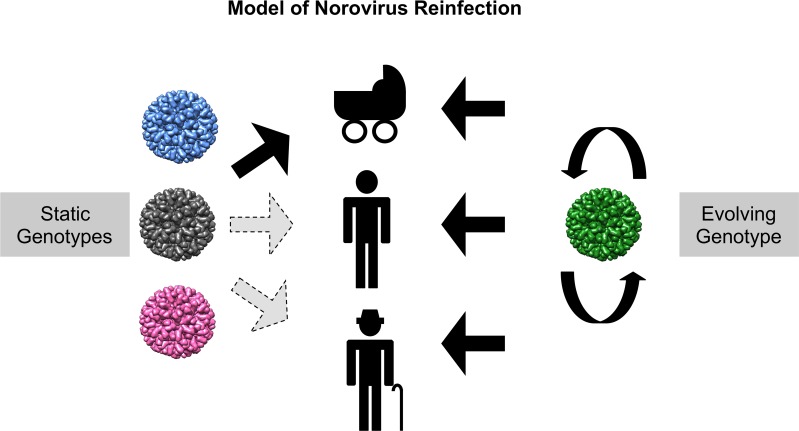
Model of norovirus evolution and infection. GII.4 noroviruses acquire phenotypic changes in their major capsid protein over time (evolving genotype), while non-GII.4 viruses retain a highly conserved capsid protein for decades (static genotype). Clustering of the different noroviruses has shown the presence of twelve groups (provisionally called immunotypes), with only one immunotype containing an evolving genotype (GII.4). Immunotypes represented by static genotypes can only re-infect individuals naïve to that particular immunotype, while the GII.4 evolving genotype can re-infect individuals by periodically replacing its variants. This model predicts that children are constantly exposed and infected with strains from each of the different immunotypes (until a broad immunity develops), while older individuals are more likely to become ill from evolving genotypes. This model would explain the epidemiological differences reported in the distribution of norovirus genotypes in children and adults [17, 21, 37, 38].

How do “static” genotypes prevail in the human population, in the face of limited antigenic diversity within the genotype? To address this question, genotypes were grouped together based on phylogenetic clustering and aa differences in their capsid proteins. These groups, or “immunotypes,” were applied to the interpretation of epidemiological observations. When examining data from a birth cohort study, or reports where children and adults were followed for years to study norovirus re-infections, genotypes belonging to the same immunotype generally did not re-infect these individuals. Thus, most of these individuals were re-infected with a varying series of genotypes (predominantly containing combinations of GII.4, GII.6, GII.3, GII.17 or GII.2), but all of them belonging to different immunotypes as defined in Fig 5C. The exception to this was the GII.4 strains in immunotype G, in which a few re-infections were observed, albeit with different GII.4 variants. Based on these data, we propose a model for norovirus re-infection in which naïve children are constantly exposed and infected with strains from each of the different immunotypes until a broad immunity develops. In contrast, older individuals (i.e. older children and adults) are more likely to become ill from evolving genotypes, as they have already acquired immunity against a number of static genotypes (Fig 6). This model not only explains the differences in the genotype distribution often seen when comparing children and adult populations [17, 37, 38], but also suggests that immunity against norovirus may be longer than initially suggested [39, 42].

For decades understanding of norovirus immunity was based on human volunteer challenge studies and animal models or *in vitro* surrogates of neutralization tests [27, 39, 71, 72]. Initial cross-challenge studies, conducted in the 1970s using the prototype GI.1 Norwalk virus and GII.1 Hawaii virus, showed a lack of protection between these two genogroups [39]. Further epidemiological data and *in vitro* assays, such as antibody blockage of carbohydrate binding to VLPs, suggested a role for immunity against the different intra-genotypic variants of GII.4 [27, 33, 58]. Norovirus vaccines are currently based on the premise to include at least two major antigens for noroviruses representing GI and GII [29, 30, 71, 73, 74]. However, recent data indicating that certain genotype-specific immune responses were unable to confer natural protection against disease raised concerns that a prohibitive number of components (almost 30) might be needed in a norovirus vaccine [17, 18, 35, 38]. Although additional studies will be needed to confirm the existence of shared antigenic groups among the norovirus genotypes, preferably by neutralization assays or animal models, our analysis provides a new perspective on the genetic and antigenic diversity of noroviruses that could lead to the identification of cross-protective strains and inform vaccine design.

## Materials and Methods

### Ethics statement

Stool specimens from the child were obtained with the written informed consent of the parent, and enrollment in National Institutes of Health (NIH) clinical study NCT01306084. Archival stool samples stored in the Laboratory of Infectious Diseases Calicivirus Repository were waived as exempt from IRB review by the NIH Office of Human Subjects Research and Protection (OHSRP 11833). Epidemiological information relating to the sample collection has been published elsewhere [18, 32, 35, 49].

### Data mining and genomics

The full-length (or nearly full-length) ORF2 sequences (encoding for VP1) from each of the 31 genotypes described for GI and GII were retrieved from GenBank (accessed on March 2015) for analyses. Alignments were performed with Clustal W as implemented in MEGA v6 [75]. Sequences from each genotype were aligned separately to minimize the presence of insertions or deletions (indels), which can arise when different genotypes are compared. Phylogenetic trees were constructed using Kimura 2-parameter as method of nt substitution and Neighbor-Joining as algorithm of reconstruction as implemented in MEGA v6 with default settings. Phylogenetic trees that used aa sequences were reconstructed using a Poisson method of aa substitution. Bootstrap analyses were used to support the clustering of the variants. Information on the strains used for the phylogenetic analyses is provided in S4 Table. Evolutionary rates (nt substitutions/site/year) for each genotype were estimated using the ORF2 sequences and the Bayesian Markov Chain Monte Carlo (MCMC) approach as implemented in the BEAST package [76]. For each set of data the General Time Reversible (GTR) model with gamma rate distribution and invariable sites parameter was used and the MCMC was run for a sufficient number of generations to reach convergence of all parameters. All evolutionary rates were calculated using strict clock model and coalescent constant size tree prior, except for genotypes GI.4, GI.6, GII.14 and GII.16, which reached convergence using Bayesian Skyline and random local clocks. Selection pressures acting in the VP1 sequences were investigated by estimating the mean rate of nonsynonymous substitutions (dN) and synonymous substitutions (dN) and the dN/dS ratio as implemented in MEGA v6. The nearly full-length genome sequences from 151 GII.4 viruses detected in Japan during 2006–2009 [50, 51] were downloaded from GenBank and analyzed using MEGA v6 and Prism software (GraphPad Prism version 7).

### Heat map plots of genotypic diversity

To visualize the aa substitutions within each genotype, a Python script (available upon request) was developed to calculate the number of aa differences and the isolation year differences between two individual strains. Isolation years were extracted from strain descriptions. The difference values were added into a matrix where the y-axis represents the isolation year differences and the x-axis the amino acid differences. Note that some cells will present more than one comparison, since strain pairs presenting the same number of aa differences and the same year difference, despite the years detected, will be included in the same cell. Heat map plots were calculated for each genotype using GraphPad Prism version 7 (GraphPad Software, La Jolla California USA), with the values representing the number of strains compared.

### Full-length genome amplification

A platform was developed to analyze the plasticity of norovirus genotypes at the full genome level. Briefly, viral RNA was extracted from 10% (w/v) stool suspensions using the MagMax Viral RNA Isolation Kit (Ambion, California, USA) following manufacturer’s recommendations. Complementary DNA was synthesized from the viral RNA using the Tx30SXN primer (GACTAGTTCTAGATCGCGAGCGGCCGCCCTTTTTTTTTTTTTTTTTTTTTTTTTTTTTT [77]) at 5μM final concentration, and the Maxima H Minus First Strand cDNA Synthesis Kit (Thermo Fisher Scientific, California, USA) following manufacturer’s recommendations except that only 0.1 μL of Enzyme Mix was used per reaction. Amplification of the full-length genome was performed using 5 μl of the RT reaction, a set of primers that target the conserved regions of the 5’- and 3’-end of GII noroviruses (GII1-35: GTGAATGAAGATGGCGTCTAACGACGCTTCCGCTG, and Tx30SXN), and the SequalPrep Long PCR Kit (Invitrogen, California, USA) following manufacturer’s recommendations. Amplicons were excised from an agarose gel and purified with the QIAquick Gel Extraction Kit (Qiagen, California, USA).

### Next-generation sequencing

Ion Torrent libraries were prepared by using 300–500 ng of full-length genome PCR amplicons following standard Ion Torrent library prep protocol. DNA was fragmented followed by the introduction of ligation barcode adapters. Adapted-ligated libraries were amplified using 13 PCR cycles, and size selected from agarose gels. Final libraries were quantified by Qubit (Invitrogen, California, USA), Bioanalyzer (Agilent), and qPCR. Libraries were normalized to 1nM, pooled at an equal molar ratio, and loaded onto a 318 v2 Chip in an Ion OneTouch2 machine. The sample from Ion OneTouch2 was transferred to an Ion OneTouch ES and then to an Ion PGM for sequencing with a 400bp kit (Life Technologies, California, USA). Ion Torrent sequence reads were de-multiplexed, and each individual set of reads was aligned to reference sequences using Bowtie2 and SAMtools [78, 79]. Aligned reads were visualized in the Integrative Genomics Viewer (IGV) [80] for single nt polymorphisms (SNPs) identification. Consensus sequence for each full-length genome was calculated using IGV. Read coverage (reads/nt position) was calculated using the genomecov command from BEDTools [81]. Sequence analyses were performed using MEGA v6 and Sequencher 5.4 (Gene Codes Corporation, Michigan, USA). The consensus sequence was calculated using default settings in Sequencher v5.4, and genomic sequences determined in this study were deposited into GenBank under Accession numbers KY424328 through KY424350. All other relevant data are within the paper and its Supporting Information files.

## Supporting Information

S1 Fig Diversity in GII.12 viruses. (a) Phylogenetic trees showing the relationship of the different strains from the GII.12 genotype. Three variants (Clusters A-C) can be discriminated when nucleotides were used for tree reconstruction. No discrete variant was detected when amino acids were used. Trees were constructed using sequences encoding the VP1 and Neighbor-Joining method as implemented in MEGA v6. (b) Diversity plot showing the accumulation of amino acid mutations over time in the VP1 from GII.12 viruses. Despite accumulation of mutations, the genotype did not diversify into different variants (dashed line representing the cut-off value for variant designation). Heat map represents the number of pairwise comparisons, red being the highest and green the lowest number of pairwise comparisons.
(TIF)

S2 Fig Diversity in GII.17 viruses. (a) Phylogenetic trees showing the relationship of the different strains from the GII.17 genotype. Four variants (Clusters A-D) were detected. Trees were constructed using sequences encoding the VP1 and Neighbor-Joining method as implemented in MEGA v6. (b) Diversity plot showing the accumulation of amino acid mutations over time in the VP1 from GII.17 viruses. Dashed line represents the cut-off value for variant designation. Heat map represents the number of pairwise comparisons, red being the highest and green the lowest number of pairwise comparisons.
(TIF)

S3 Fig Accumulation of mutations in VP1 during the shedding phase of an acute GII.4 infection in an immunocompetent individual. Four amino acid mutations at days 14 and 21 mapped into three major (A, C, and E) antigenic sites of GII.4 noroviruses. The GII.4 norovirus P domain was rendered using the previously solved crystal structure of norovirus VA387 (pdb file: 2OBR), and the UCSF Chimera package (http://www.rbvi.ucsf.edu/chimera).
(TIFF)

S4 Fig Accumulation of mutations in GII.4 Den_Haag variants detected in Japan. (a) Full-length genome analyses showing the accumulation of nucleotide (upper figure) and amino acid (lower figure) mutations during three consecutive seasons in Japan [50, 51]. (b) Data from individual open reading frames (ORF) shows that ORF2 (encoding VP1) presents the lowest number of amino acid accumulations, which is in concordance with data obtained using next-generation sequencing for the Maryland nursing homes outbreaks in Fig 4.
(TIFF)

S5 Fig Trees inferred by using the Maximum Likelihood method based on the Tamura-Nei model presented topologies similar to that shown by the Neighbor-joining method in Fig 5. (TIF)

S6 Fig Matrix showing the frequency of re-infection and the genotypes detected. Data was obtained from studies that followed the natural history of norovirus infection [17, 18, 22, 38, 52–54]. Every possible combination was recorded from the re-infection cases. Re-infection with strains from the same immunotype are indicated by black cells. For immunotype designation of each norovirus genotype refer to Fig 5.
(TIF)

S1 Table Average reads per nucleotide position obtained for the twenty-four nearly full-length norovirus genomes analyzed by next-generation sequencing in this study. (DOC)

S2 Table Nucleotide mutations observed during the intra-host evolution of norovirus genotypes GII.4, GII.6 and GII.17 and resulting amino acid substitutions. (DOC)

S3 Table Norovirus ORF2 sequences used in the construction of trees representing the clustering of immunotypes. (DOCX)

S4 Table Sequences of GII.6, GII.12 and GII.17 noroviruses used in this study. (DOCX)

## References

[1] Sanjuan R, Nebot MR, Chirico N, Mansky LM, Belshaw R. Viral mutation rates. J Virol. 2010;84(19):9733–48. PubMed Central PMCID: PMC2937809. 10.1128/JVI.00694-10 20660197 PMC2937809

[2] Drake JW. Rates of spontaneous mutation among RNA viruses. Proc Natl Acad Sci U S A. 1993;90(9):4171–5. PubMed Central PMCID: PMC46468. 8387212 10.1073/pnas.90.9.4171 PMC46468

[3] Duffy S, Shackelton LA, Holmes EC. Rates of evolutionary change in viruses: patterns and determinants. Nat Rev Genet. 2008;9(4):267–76. 10.1038/nrg2323 18319742

[4] Worobey M, Holmes EC. Evolutionary aspects of recombination in RNA viruses. J Gen Virol. 1999;80 (Pt 10):2535–43. 10573145 10.1099/0022-1317-80-10-2535

[5] Grenfell BT, Pybus OG, Gog JR, Wood JL, Daly JM, Mumford JA, et al Unifying the epidemiological and evolutionary dynamics of pathogens. Science. 2004;303(5656):327–32. 10.1126/science.1090727 14726583

[6] Smith DJ, Lapedes AS, de Jong JC, Bestebroer TM, Rimmelzwaan GF, Osterhaus AD, et al Mapping the antigenic and genetic evolution of influenza virus. Science. 2004;305(5682):371–6. 10.1126/science.1097211 15218094

[7] Koelle K, Cobey S, Grenfell B, Pascual M. Epochal evolution shapes the phylodynamics of interpandemic influenza A (H3N2) in humans. Science. 2006;314(5807):1898–903. 10.1126/science.1132745 17185596

[8] Vignuzzi M, Stone JK, Arnold JJ, Cameron CE, Andino R. Quasispecies diversity determines pathogenesis through cooperative interactions in a viral population. Nature. 2006;439(7074):344–8. PubMed Central PMCID: PMC1569948. 10.1038/nature04388 16327776 PMC1569948

[9] Trento A, Abrego L, Rodriguez-Fernandez R, Gonzalez-Sanchez MI, Gonzalez-Martinez F, Delfraro A, et al Conservation of G-Protein Epitopes in Respiratory Syncytial Virus (Group A) Despite Broad Genetic Diversity: Is Antibody Selection Involved in Virus Evolution? J Virol. 2015;89(15):7776–85. PubMed Central PMCID: PMC4505632. 10.1128/JVI.00467-15 25995258 PMC4505632

[10] Payne DC, Vinje J, Szilagyi PG, Edwards KM, Staat MA, Weinberg GA, et al Norovirus and medically attended gastroenteritis in U.S. children. The New England journal of medicine. 2013;368(12):1121–30. 10.1056/NEJMsa1206589 23514289 PMC4618551

[11] Bucardo F, Reyes Y, Svensson L, Nordgren J. Predominance of norovirus and sapovirus in Nicaragua after implementation of universal rotavirus vaccination. PLoS One. 2014;9(5):e98201 PubMed Central PMCID: PMC4029982. 10.1371/journal.pone.0098201 24849288 PMC4029982

[12] McAtee CL, Webman R, Gilman RH, Mejia C, Bern C, Apaza S, et al Burden of Norovirus and Rotavirus in Children After Rotavirus Vaccine Introduction, Cochabamba, Bolivia. Am J Trop Med Hyg. 2016;94(1):212–7. PubMed Central PMCID: PMC4710432. 10.4269/ajtmh.15-0203 26598569 PMC4710432

[13] Hemming M, Rasanen S, Huhti L, Paloniemi M, Salminen M, Vesikari T. Major reduction of rotavirus, but not norovirus, gastroenteritis in children seen in hospital after the introduction of RotaTeq vaccine into the National Immunization Programme in Finland. Eur J Pediatr. 2013;172(6):739–46. 10.1007/s00431-013-1945-3 23361964 PMC7086648

[14] Patel MM, Widdowson MA, Glass RI, Akazawa K, Vinje J, Parashar UD. Systematic literature review of role of noroviruses in sporadic gastroenteritis. Emerg Infect Dis. 2008;14(8):1224–31. Epub 2008/08/06. PubMed Central PMCID: PMC2600393. 10.3201/eid1408.071114 18680645 PMC2600393

[15] Lanata CF, Fischer-Walker CL, Olascoaga AC, Torres CX, Aryee MJ, Black RE, et al Global causes of diarrheal disease mortality in children <5 years of age: a systematic review. PLoS One. 2013;8(9):e72788 PubMed Central PMCID: PMC3762858. 10.1371/journal.pone.0072788 24023773 PMC3762858

[16] O'Ryan ML, Lucero Y, Prado V, Santolaya ME, Rabello M, Solis Y, et al Symptomatic and asymptomatic rotavirus and norovirus infections during infancy in a Chilean birth cohort. Pediatr Infect Dis J. 2009;28(10):879–84. 10.1097/INF.0b013e3181a4bb60 19672213

[17] Saito M, Goel-Apaza S, Espetia S, Velasquez D, Cabrera L, Loli S, et al Multiple Norovirus Infections in a Birth Cohort in a Peruvian Periurban Community. Clinical infectious diseases: an official publication of the Infectious Diseases Society of America. 2013. 10.1093/cid/cit763 PMC3905757 24300042

[18] Parra GI, Green KY. Sequential gastroenteritis episodes caused by 2 norovirus genotypes. Emerg Infect Dis. 2014;20(6):1016–8. PubMed Central PMCID: PMC4036768. 10.3201/eid2006.131627 24857806 PMC4036768

[19] Teunis PF, Sukhrie FH, Vennema H, Bogerman J, Beersma MF, Koopmans MP. Shedding of norovirus in symptomatic and asymptomatic infections. Epidemiol Infect. 2015;143(8):1710–7. 10.1017/S095026881400274X 25336060 PMC9507237

[20] Green KY. Norovirus infection in immunocompromised hosts. Clin Microbiol Infect. 2014;20(8):717–23. 10.1111/1469-0691.12761 25040790 PMC11036326

[21] Boon D, Mahar JE, Abente EJ, Kirkwood CD, Purcell RH, Kapikian AZ, et al Comparative evolution of GII.3 and GII.4 norovirus over a 31-year period. J Virol. 2011;85(17):8656–66. PubMed Central PMCID: PMC3165818. 10.1128/JVI.00472-11 21715504 PMC3165818

[22] Chan MC, Lee N, Hung TN, Kwok K, Cheung K, Tin EK, et al Rapid emergence and predominance of a broadly recognizing and fast-evolving norovirus GII.17 variant in late 2014. Nat Commun. 2015;6:10061 PubMed Central PMCID: PMC4686777. 10.1038/ncomms10061 26625712 PMC4686777

[23] Cuevas JM, Combe M, Torres-Puente M, Garijo R, Guix S, Buesa J, et al Human norovirus hyper-mutation revealed by ultra-deep sequencing. Infect Genet Evol. 2016;41:233–9. 10.1016/j.meegid.2016.04.017 27094861 PMC7172324

[24] de Graaf M, van Beek J, Koopmans MP. Human norovirus transmission and evolution in a changing world. Nat Rev Microbiol. 2016;14(7):421–33. 10.1038/nrmicro.2016.48 27211790

[25] Kroneman A, Vega E, Vennema H, Vinje J, White PA, Hansman G, et al Proposal for a unified norovirus nomenclature and genotyping. Arch Virol. 2013;158(10):2059–68. 10.1007/s00705-013-1708-5 23615870 PMC5570552

[26] Prasad BV, Hardy ME, Dokland T, Bella J, Rossmann MG, Estes MK. X-ray crystallographic structure of the Norwalk virus capsid. Science. 1999;286(5438):287–90. Epub 1999/10/09. 10514371 10.1126/science.286.5438.287

[27] Lindesmith LC, Donaldson EF, Lobue AD, Cannon JL, Zheng DP, Vinje J, et al Mechanisms of GII.4 norovirus persistence in human populations. PLoS Med. 2008;5(2):e31 Epub 2008/02/15. PubMed Central PMCID: PMC2235898. 10.1371/journal.pmed.0050031 18271619 PMC2235898

[28] Reeck A, Kavanagh O, Estes MK, Opekun AR, Gilger MA, Graham DY, et al Serological correlate of protection against norovirus-induced gastroenteritis. J Infect Dis. 2010;202(8):1212–8. Epub 2010/09/08. PubMed Central PMCID: PMC2945238. 10.1086/656364 20815703 PMC2945238

[29] Parra GI, Bok K, Taylor R, Haynes JR, Sosnovtsev SV, Richardson C, et al Immunogenicity and specificity of norovirus Consensus GII.4 virus-like particles in monovalent and bivalent vaccine formulations. Vaccine. 2012;30(24):3580–6. Epub 2012/04/04. PubMed Central PMCID: PMC3359014. 10.1016/j.vaccine.2012.03.050 22469864 PMC3359014

[30] Richardson C, Bargatze RF, Goodwin R, Mendelman PM. Norovirus virus-like particle vaccines for the prevention of acute gastroenteritis. Expert review of vaccines. 2013;12(2):155–67. 10.1586/erv.12.145 23414407

[31] Vinje J. Advances in laboratory methods for detection and typing of norovirus. J Clin Microbiol. 2015;53(2):373–81. PubMed Central PMCID: PMC4298492. 10.1128/JCM.01535-14 24989606 PMC4298492

[32] Green KY, Belliot G, Taylor JL, Valdesuso J, Lew JF, Kapikian AZ, et al A predominant role for Norwalk-like viruses as agents of epidemic gastroenteritis in Maryland nursing homes for the elderly. J Infect Dis. 2002;185(2):133–46. PubMed Central PMCID: PMC4793396. 10.1086/338365 11807686 PMC4793396

[33] Siebenga JJ, Vennema H, Zheng DP, Vinje J, Lee BE, Pang XL, et al Norovirus illness is a global problem: emergence and spread of norovirus GII.4 variants, 2001–2007. J Infect Dis. 2009;200(5):802–12. 10.1086/605127 19627248

[34] Lu J, Sun L, Fang L, Yang F, Mo Y, Lao J, et al Gastroenteritis outbreaks caused by norovirus GII.17, Guangdong Province, China, 2014–2015. Emerg Infect Dis. 2015;(7). 10.3201/eid2107.150226 PMC4480401 26080037

[35] Parra GI, Green KY. Genome of Emerging Norovirus GII.17, United States, 2014. Emerg Infect Dis. 2015;21(8):1477–9. PubMed Central PMCID: PMC4517714. 10.3201/eid2108.150652 26196235 PMC4517714

[36] Matsushima Y, Ishikawa M, Shimizu T, Komane A, Kasuo S, Shinohara M, et al Genetic analyses of GII.17 norovirus strains in diarrheal disease outbreaks from December 2014 to March 2015 in Japan reveal a novel polymerase sequence and amino acid substitutions in the capsid region. Euro surveillance: bulletin europeen sur les maladies transmissibles = European communicable disease bulletin. 2015;20(26). 10.2807/1560-7917.es2015.20.26.21173 26159307

[37] Zhirakovskaia EV, Tikunov AY, Bodnev SA, Klemesheva VV, Netesov SV, Tikunova NV. Molecular epidemiology of noroviruses associated with sporadic gastroenteritis in children in Novosibirsk, Russia, 2003–2012. J Med Virol. 2015;87(5):740–53. 10.1002/jmv.24068 25693507

[38] Sakon N, Yamazaki K, Nakata K, Kanbayashi D, Yoda T, Mantani M, et al Impact of genotype-specific herd immunity on the circulatory dynamism of norovirus: a 10-year longitudinal study of viral acute gastroenteritis. J Infect Dis. 2015;211(6):879–88. 10.1093/infdis/jiu496 25210139

[39] Wyatt RG, Dolin R, Blacklow NR, DuPont HL, Buscho RF, Thornhill TS, et al Comparison of three agents of acute infectious nonbacterial gastroenteritis by cross-challenge in volunteers. J Infect Dis. 1974;129(6):709–14. Epub 1974/06/01. 4209723 10.1093/infdis/129.6.709

[40] Czako R, Atmar RL, Opekun AR, Gilger MA, Graham DY, Estes MK. Experimental human infection with Norwalk virus elicits a surrogate neutralizing antibody response with cross-genogroup activity. Clin Vaccine Immunol. 2015;22(2):221–8. PubMed Central PMCID: PMC4308873. 10.1128/CVI.00516-14 25540269 PMC4308873

[41] Glass RI, Parashar UD, Estes MK. Norovirus gastroenteritis. The New England journal of medicine. 2009;361(18):1776–85. PubMed Central PMCID: PMC3880795. 10.1056/NEJMra0804575 19864676 PMC3880795

[42] Simmons K, Gambhir M, Leon J, Lopman B. Duration of immunity to norovirus gastroenteritis. Emerg Infect Dis. 2013;19(8):1260–7. PubMed Central PMCID: PMC3739512. 10.3201/eid1908.130472 23876612 PMC3739512

[43] de Graaf M, van Beek J, Vennema H, Podkolzin AT, Hewitt J, Bucardo F, et al Emergence of a novel GII.17 norovirus—End of the GII.4 era? Euro surveillance: bulletin europeen sur les maladies transmissibles = European communicable disease bulletin. 2015;20(26). 10.2807/1560-7917.es2015.20.26.21178 PMC5921880 26159308

[44] Lu J, Fang L, Zheng H, Lao J, Yang F, Sun L, et al The Evolution and Transmission of Epidemic GII.17 Noroviruses. J Infect Dis. 2016;214(4):556–64. PubMed Central PMCID: PMC4957445. 10.1093/infdis/jiw208 27354370 PMC4957445

[45] Woelk CH, Pybus OG, Jin L, Brown DW, Holmes EC. Increased positive selection pressure in persistent (SSPE) versus acute measles virus infections. J Gen Virol. 2002;83(Pt 6):1419–30. 10.1099/0022-1317-83-6-1419 12029157

[46] Holmes EC, Woelk CH, Kassis R, Bourhy H. Genetic constraints and the adaptive evolution of rabies virus in nature. Virology. 2002;292(2):247–57. 10.1006/viro.2001.1271 11878928

[47] Hanada K, Suzuki Y, Gojobori T. A large variation in the rates of synonymous substitution for RNA viruses and its relationship to a diversity of viral infection and transmission modes. Molecular biology and evolution. 2004;21(6):1074–80. 10.1093/molbev/msh109 15014142 PMC7107514

[48] Debbink K, Lindesmith LC, Donaldson EF, Baric RS. Norovirus immunity and the great escape. PLoS pathogens. 2012;8(10):e1002921 PubMed Central PMCID: PMC3475665. 10.1371/journal.ppat.1002921 23093932 PMC3475665

[49] Buscho R, Wyatt RG, Dolin R, Blacklow NR, DuPont HL, Chanock RM. Recurrent institutional outbreaks of acute infectious nonbacterial gastroenteritis: epidemiology and etiology. Am J Epidemiol. 1973;98(3):192–8. 4741840 10.1093/oxfordjournals.aje.a121548

[50] Motomura K, Oka T, Yokoyama M, Nakamura H, Mori H, Ode H, et al Identification of monomorphic and divergent haplotypes in the 2006–2007 norovirus GII/4 epidemic population by genomewide tracing of evolutionary history. J Virol. 2008;82(22):11247–62. PubMed Central PMCID: PMC2573252. 10.1128/JVI.00897-08 18768979 PMC2573252

[51] Motomura K, Yokoyama M, Ode H, Nakamura H, Mori H, Kanda T, et al Divergent evolution of norovirus GII/4 by genome recombination from May 2006 to February 2009 in Japan. J Virol. 2010;84(16):8085–97. PubMed Central PMCID: PMC2916515. 10.1128/JVI.02125-09 20534859 PMC2916515

[52] Kirkwood CD, Streitberg R. Calicivirus shedding in children after recovery from diarrhoeal disease. J Clin Virol. 2008;43(3):346–8. 10.1016/j.jcv.2008.08.001 18789755

[53] Takanashi S, Wang Q, Chen N, Shen Q, Jung K, Zhang Z, et al Characterization of emerging GII.g/GII.12 noroviruses from a gastroenteritis outbreak in the United States in 2010. J Clin Microbiol. 2011;49(9):3234–44. PubMed Central PMCID: PMC3165588. 10.1128/JCM.00305-11 21752978 PMC3165588

[54] Blazevic V, Malm M, Salminen M, Oikarinen S, Hyoty H, Veijola R, et al Multiple consecutive norovirus infections in the first 2 years of life. Eur J Pediatr. 2015;174(12):1679–83. PubMed Central PMCID: PMC4662724. 10.1007/s00431-015-2591-8 26152345 PMC4662724

[55] Jenkins GM, Rambaut A, Pybus OG, Holmes EC. Rates of molecular evolution in RNA viruses: a quantitative phylogenetic analysis. J Mol Evol. 2002;54(2):156–65. 10.1007/s00239-001-0064-3 11821909

[56] Fonager J, Hindbaek L, Fischer T. Rapid emergence and antigenic diversification of the norovirus 2012 Sydney variant in Denmark, October to December, 2012. Euro surveillance: bulletin europeen sur les maladies transmissibles = European communicable disease bulletin. 2013;18(9). 23470017

[57] Bok K, Abente EJ, Realpe-Quintero M, Mitra T, Sosnovtsev SV, Kapikian AZ, et al Evolutionary dynamics of GII.4 noroviruses over a 34-year period. J Virol. 2009;83(22):11890–901. Epub 2009/09/18. PubMed Central PMCID: PMC2772697. 10.1128/JVI.00864-09 19759138 PMC2772697

[58] Siebenga JJ, Lemey P, Kosakovsky Pond SL, Rambaut A, Vennema H, Koopmans M. Phylodynamic reconstruction reveals norovirus GII.4 epidemic expansions and their molecular determinants. PLoS pathogens. 2010;6(5):e1000884 PubMed Central PMCID: PMC2865530. 10.1371/journal.ppat.1000884 20463813 PMC2865530

[59] Rackoff LA, Bok K, Green KY, Kapikian AZ. Epidemiology and evolution of rotaviruses and noroviruses from an archival WHO Global Study in Children (1976–79) with implications for vaccine design. PLoS One. 2013;8(3):e59394 PubMed Central PMCID: PMC3607611. 10.1371/journal.pone.0059394 23536875 PMC3607611

[60] Fioretti JM, Bello G, Rocha MS, Victoria M, Leite JP, Miagostovich MP. Temporal dynamics of norovirus GII.4 variants in Brazil between 2004 and 2012. PLoS One. 2014;9(3):e92988 PubMed Central PMCID: PMC3965504. 10.1371/journal.pone.0092988 24667283 PMC3965504

[61] Kobayashi M, Yoshizumi S, Kogawa S, Takahashi T, Ueki Y, Shinohara M, et al Molecular Evolution of the Capsid Gene in Norovirus Genogroup I. Sci Rep. 2015;5:13806 PubMed Central PMCID: PMC4559769. 10.1038/srep13806 26338545 PMC4559769

[62] Jorba J, Campagnoli R, De L, Kew O. Calibration of multiple poliovirus molecular clocks covering an extended evolutionary range. J Virol. 2008;82(9):4429–40. PubMed Central PMCID: PMC2293050. 10.1128/JVI.02354-07 18287242 PMC2293050

[63] Xue L, Wu Q, Kou X, Cai W, Zhang J, Guo W. Genome characterization of a GII.6 norovirus strain identified in China. Infect Genet Evol. 2015;31:110–7. 10.1016/j.meegid.2015.01.027 25660038

[64] Debbink K, Lindesmith LC, Donaldson EF, Costantini V, Beltramello M, Corti D, et al Emergence of new pandemic GII.4 Sydney norovirus strain correlates with escape from herd immunity. J Infect Dis. 2013;208(11):1877–87. PubMed Central PMCID: PMC3814837. 10.1093/infdis/jit370 23908476 PMC3814837

[65] Bodhidatta L, Abente E, Neesanant P, Nakjarung K, Sirichote P, Bunyarakyothin G, et al Molecular epidemiology and genotype distribution of noroviruses in children in Thailand from 2004 to 2010: a multi-site study. J Med Virol. 2015;87(4):664–74. 10.1002/jmv.24108 25649836

[66] Wang YH, Zhou DJ, Zhou X, Yang T, Ghosh S, Pang BB, et al Molecular epidemiology of noroviruses in children and adults with acute gastroenteritis in Wuhan, China, 2007–2010. Arch Virol. 2012;157(12):2417–24. 10.1007/s00705-012-1437-1 22886184

[67] Eden JS, Tanaka MM, Boni MF, Rawlinson WD, White PA. Recombination within the pandemic norovirus GII.4 lineage. J Virol. 2013;87(11):6270–82. PubMed Central PMCID: PMC3648122. 10.1128/JVI.03464-12 23536665 PMC3648122

[68] Bull RA, Eden JS, Luciani F, McElroy K, Rawlinson WD, White PA. Contribution of intra- and interhost dynamics to norovirus evolution. J Virol. 2012;86(6):3219–29. PubMed Central PMCID: PMC3302298. 10.1128/JVI.06712-11 22205753 PMC3302298

[69] Vega E, Donaldson E, Huynh J, Barclay L, Lopman B, Baric R, et al RNA populations in immunocompromised patients as reservoirs for novel norovirus variants. J Virol. 2014;88(24):14184–96. PubMed Central PMCID: PMC4249157. 10.1128/JVI.02494-14 25275120 PMC4249157

[70] Lauring AS, Frydman J, Andino R. The role of mutational robustness in RNA virus evolution. Nat Rev Microbiol. 2013;11(5):327–36. PubMed Central PMCID: PMC3981611. 10.1038/nrmicro3003 23524517 PMC3981611

[71] Atmar RL, Bernstein DI, Lyon GM, Treanor JJ, Al-Ibrahim MS, Graham DY, et al Serological Correlates of Protection against a GII.4 Norovirus. Clin Vaccine Immunol. 2015;22(8):923–9. PubMed Central PMCID: PMC4519714. 10.1128/CVI.00196-15 26041041 PMC4519714

[72] Czako R, Atmar RL, Opekun AR, Gilger MA, Graham DY, Estes MK. Serum hemagglutination inhibition activity correlates with protection from gastroenteritis in persons infected with Norwalk virus. Clin Vaccine Immunol. 2012;19(2):284–7. PubMed Central PMCID: PMC3272916. 10.1128/CVI.05592-11 22190401 PMC3272916

[73] Treanor JJ, Atmar RL, Frey SE, Gormley R, Chen WH, Ferreira J, et al A novel intramuscular bivalent norovirus virus-like particle vaccine candidate—reactogenicity, safety, and immunogenicity in a phase 1 trial in healthy adults. J Infect Dis. 2014;210(11):1763–71. 10.1093/infdis/jiu337 24951828 PMC8483568

[74] Bernstein DI, Atmar RL, Lyon GM, Treanor JJ, Chen WH, Jiang X, et al Norovirus vaccine against experimental human GII.4 virus illness: a challenge study in healthy adults. J Infect Dis. 2015;211(6):870–8. 25210140 10.1093/infdis/jiu497 PMC5914500

[75] Tamura K, Stecher G, Peterson D, Filipski A, Kumar S. MEGA6: Molecular Evolutionary Genetics Analysis version 6.0. Molecular biology and evolution. 2013;30(12):2725–9. PubMed Central PMCID: PMC3840312. 10.1093/molbev/mst197 24132122 PMC3840312

[76] Drummond AJ, Suchard MA, Xie D, Rambaut A. Bayesian phylogenetics with BEAUti and the BEAST 1.7. Molecular biology and evolution. 2012;29(8):1969–73. PubMed Central PMCID: PMC3408070. 10.1093/molbev/mss075 22367748 PMC3408070

[77] Katayama K, Shirato-Horikoshi H, Kojima S, Kageyama T, Oka T, Hoshino F, et al Phylogenetic analysis of the complete genome of 18 Norwalk-like viruses. Virology. 2002;299(2):225–39. 12202225 10.1006/viro.2002.1568

[78] Li H, Handsaker B, Wysoker A, Fennell T, Ruan J, Homer N, et al The Sequence Alignment/Map format and SAMtools. Bioinformatics. 2009;25(16):2078–9. PubMed Central PMCID: PMC2723002. 10.1093/bioinformatics/btp352 19505943 PMC2723002

[79] Langmead B, Salzberg SL. Fast gapped-read alignment with Bowtie 2. Nat Methods. 2012;9(4):357–9. PubMed Central PMCID: PMC3322381. 10.1038/nmeth.1923 22388286 PMC3322381

[80] Thorvaldsdottir H, Robinson JT, Mesirov JP. Integrative Genomics Viewer (IGV): high-performance genomics data visualization and exploration. Brief Bioinform. 2013;14(2):178–92. PubMed Central PMCID: PMC3603213. 10.1093/bib/bbs017 22517427 PMC3603213

[81] Quinlan AR, Hall IM. BEDTools: a flexible suite of utilities for comparing genomic features. Bioinformatics. 2010;26(6):841–2. PubMed Central PMCID: PMC2832824. 10.1093/bioinformatics/btq033 20110278 PMC2832824

