# Supplementary figures and images for: Static and Evolving Norovirus Genotypes: Implications for Epidemiology and Immunity
Source: PLoS Pathog. 2017 Jan 19;13(1):e1006136. doi: 10.1371/journal.ppat.1006136 (PMC5283768; doi:10.1371/journal.ppat.1006136)

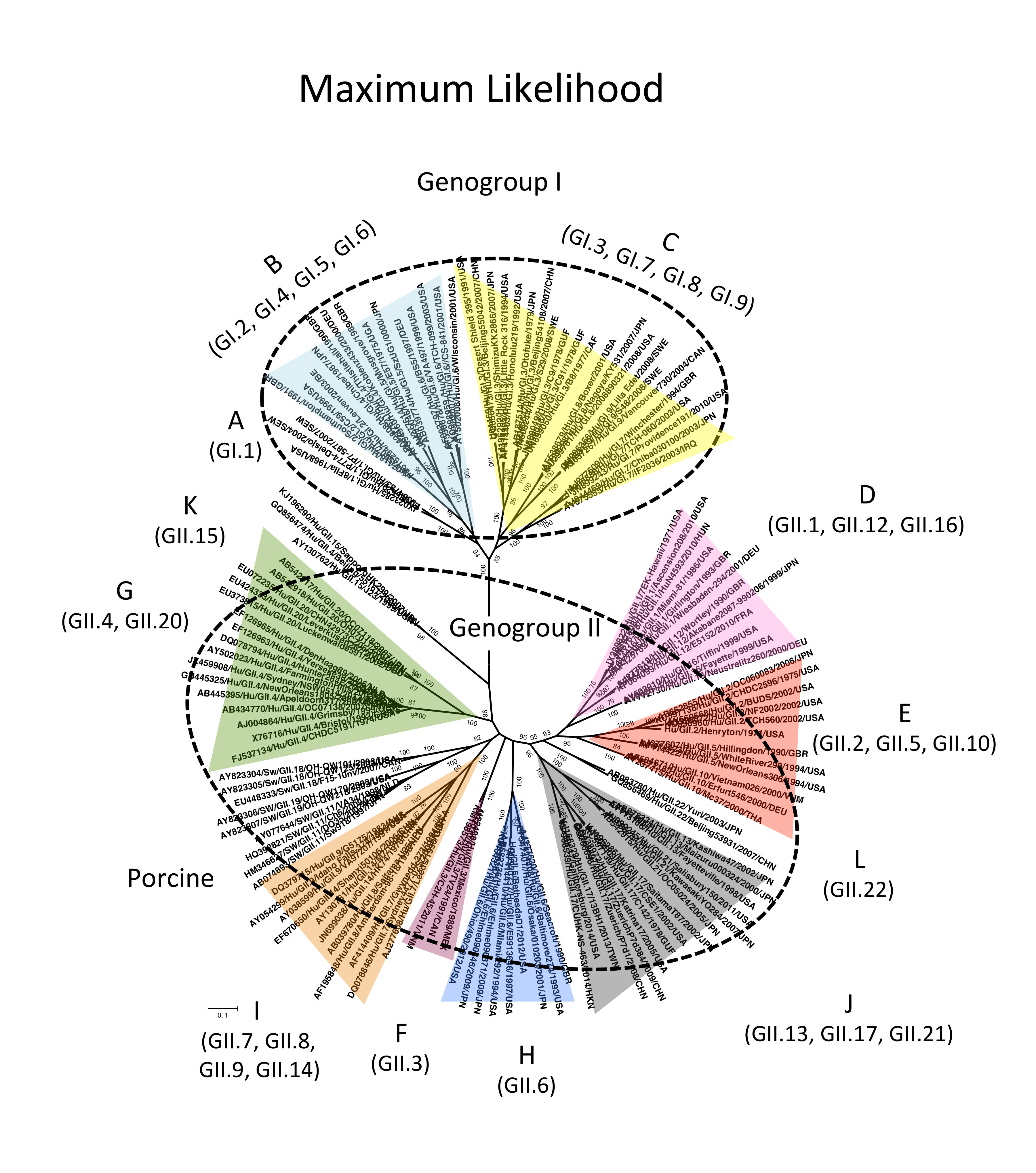

Supplement: S5 Fig — (TIF) [file ppat.1006136.s005.tif]

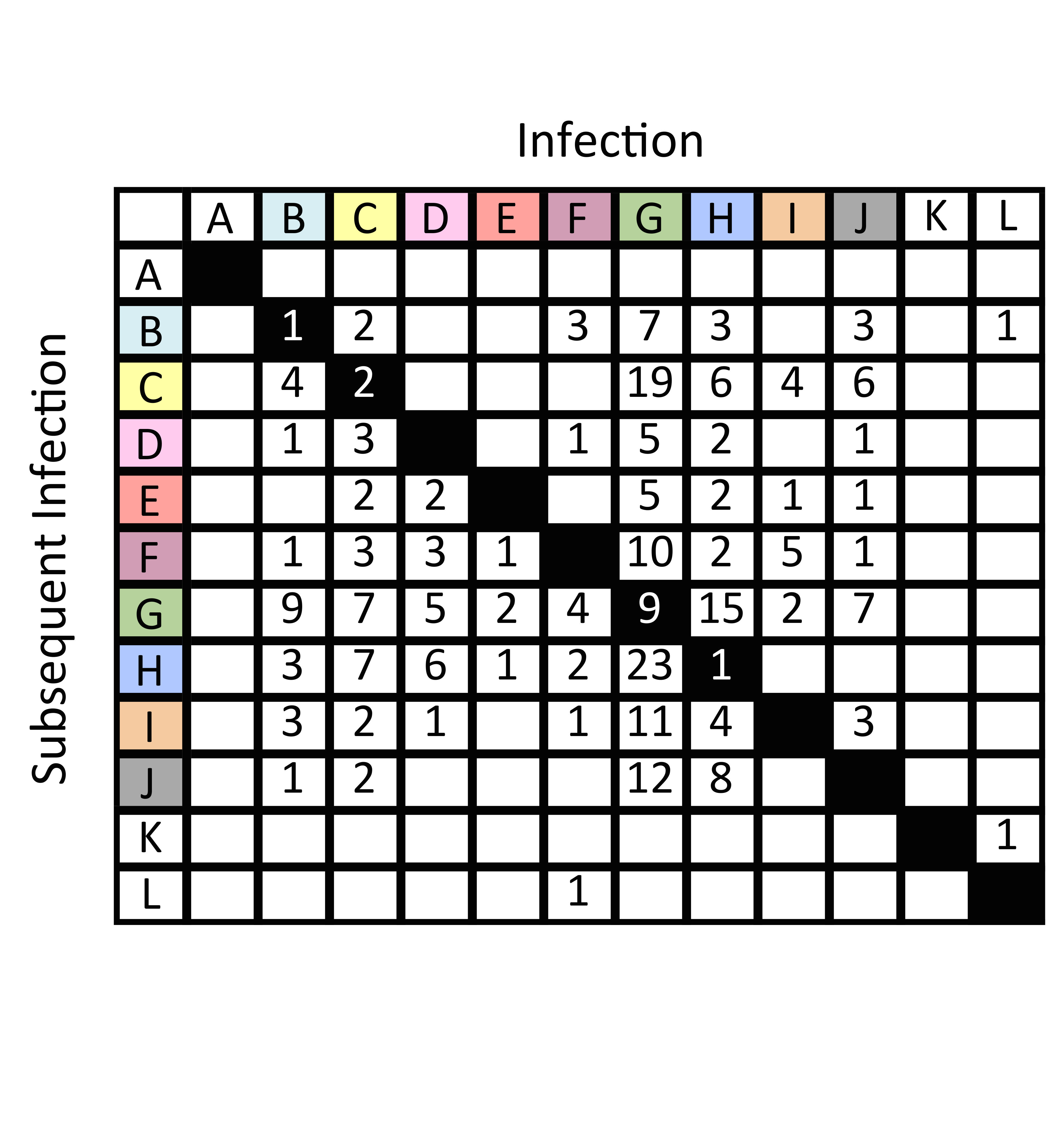

Supplement: S6 Fig — Data was obtained from studies that followed the natural history of norovirus infection [17, 18, 22, 38, 52–54]. Every possible combination was recorded from the re-infection cases. Re-infection with strains from the same immunotype are indicated by black cells. For immunotype designation of each norovirus genotype refer to Fig 5. (TIF) [file ppat.1006136.s006.tif]
