# Supplementary material for: Static and Evolving Norovirus Genotypes: Implications for Epidemiology and Immunity
Source: PLoS Pathog. 2017 Jan 19;13(1):e1006136. doi: 10.1371/journal.ppat.1006136 (PMC5283768; doi:10.1371/journal.ppat.1006136)
Supplement: S1 Table — (DOC) [file ppat.1006136.s007.doc]

**Table S1.** Norovirus genome coverage

|  | |  |  |  |
| --- | --- | --- | --- | --- |
| Sample | | | | Number of reads per nt position (Average ± SD) |
|  | |  |  |  |
| Hu/GII.4/Rockville/2012 | | | D1 | 8850 ± 3854 |
|  |  |  | D7 | 12583 ± 5617 |
|  |  |  | D14 | 24657 ± 11392 |
|  |  |  | D21 | 9617 ± 4049 |
|  | |  |  |  |
| Hu/GII.6/Bethesda/2012 | | | D1 | 28523 ± 16399 |
|  |  |  | D7 | 16167 ± 4823 |
|  |  |  | D14 | 19499 ± 6039 |
|  |  |  | D21 | 11891 ± 2928 |
|  | |  |  |  |
| Hu/GII.17/Gaithersburg/2014 | | | D1 | 10287 ± 2173 |
|  |  |  | D7 | 11571 ± 3104 |
|  |  |  | D14 | 7913 ± 2061 |
|  |  | |  |  |
| Maryland Nursing Homes Outbreak | Nov-87 | | 104-2 | 10874 ± 3327 |
|  |  |  | 104-5 | 9694 ± 2556 |
|  |  |  | 104-7 | 16872 ± 4197 |
|  |  |  | 104-8 | 11868 ± 2897 |
|  |  |  | 104-9 | 15704 ± 3836 |
|  | Dec-87 | | 143-4 | 11345 ± 2948 |
|  |  |  | 143-7 | 3114 ± 1025 |
|  | Jan-88 | | 04-1A | 3016 ± 906 |
|  |  |  | 04-7A | 12903 ± 3478 |
|  |  | |  |  |
| Henryton Hospital Outbreak | Dec-71 | | H1971 | 27976 ± 15664 |
|  |  |  | S1971 | 4582 ± 3214 |
|  |  |  | P1971 | 5159 ± 2611 |
|  |  |  | L1971 | 16445 ± 7880 |
|  |  | |  |  |
|  |  | |  |  |
