# Supplementary material for: Static and Evolving Norovirus Genotypes: Implications for Epidemiology and Immunity
Source: PLoS Pathog. 2017 Jan 19;13(1):e1006136. doi: 10.1371/journal.ppat.1006136 (PMC5283768; doi:10.1371/journal.ppat.1006136)
Supplement: S2 Table — (DOC) [file ppat.1006136.s008.doc]

Table S2. Nucleotide mutations observed during the intra-host

evolution of norovirus genotypes GII.4, GII.6 and GII.17 and resulting amino acid

substitutions.

|  |  |  |
| --- | --- | --- |
| Sample in Comparison with Day 1 | Nucleotide Position | Amino Acid Substitution |
| Hu/GII.4/RockvilleDay14/2012/US | 30 | D >>> F |
|  | 1235 | - |
|  | 1477 | - |
|  | 2094 | G >>> P |
|  | 2951 | E >>> K |
|  | 3572 | G >>> R |
|  | 5621 | - |
|  | 6187 | E >>> A |
|  | 6319 | N >>> S |
|  | 6568 | G >>> D |
|  | 7227 | T >>> I |
|  | 7451 | F >>> L |
| Hu/GII.4/RockvilleDay21/2012/US | 2585 | D >>> N |
|  | 3040 | - |
|  | 3094 | - |
|  | 3928 | - |
|  | 4027 | - |
|  | 4408 | - |
|  | 4487 | S >>> P |
|  | 4695 | T >>> I |
|  | 4987 | - |
|  | 5131 | A >>> V |
|  | 5827 | K >>> R/T |
|  | 5861 | - |
|  | 5962 | I >>> T |
|  | 6154 | D >>> G |
|  | 6213 | A >>> T |
|  | 6318 | - |
|  | 6319 | N >>> S |
|  | 6704 | - |
|  | 6706 | - |
|  | 7075 | - |
| Hu/GII.6/BethesdaDay21/2012/US | 38 | F >>> L |
|  | 1470 | T >>> I |
|  | 5594 | S >>> P |
|  | 6135 | N >>> S |
| Hu/GII.17/GaithersburgDay14/2014/US | 3333 | - |
|  | 5614 | - |
|  |  |  |

Note: “-“ represents synonymous mutation; single or mixed population of amino acid substitutions is indicated
