# Supplementary material for: Static and Evolving Norovirus Genotypes: Implications for Epidemiology and Immunity
Source: PLoS Pathog. 2017 Jan 19;13(1):e1006136. doi: 10.1371/journal.ppat.1006136 (PMC5283768; doi:10.1371/journal.ppat.1006136)
Supplement: S3 Table — (DOCX) [file ppat.1006136.s009.docx]

**Table S3.**  Norovirus ORF2 sequences used in the construction of trees representing the clustering of immunotypes.

**Noroviruses (n=131) representing the currently defined GI and GII genotypes were used for tree reconstruction (GenBank Accession number, host, genotype, strain name, collection year and country are provided)**

| **Accession** | | **Host** | **Genotype** | **Strain** | **Collection Year** | **Country** |
| --- | --- | --- | --- | --- | --- | --- |
| 1 | JX023285 | Hu | GI.1 | 8FIIa | 1968 | USA |
| 2 | EU085529 | Hu | GI.1 | P774-Delsjo | 2004 | SEW |
| 3 | FJ384783 | Hu | GI.1 | P7-587 | 2007 | SEW |
| 4 | L07418 | Hu | GI.2 | Southampton | 1991 | GBR |
| 5 | AF435807 | Hu | GI.2 | C59 | 1999 | USA |
| 6 | FJ515294 | Hu | GI.2 | Leuven | 2003 | BEL |
| 7 | U04469 | Hu | GI.3 | Desert_Shield_395 | 1991 | USA |
| 8 | GQ856473 | Hu | GI.3 | Beijing55042 | 2007 | CHN |
| 9 | KJ196292 | Hu | GI.3 | ShimizuKK2866 | 2007 | JPN |
| 10 | AF414405 | Hu | GI.3 | Little_Rock_316 | 1994 | USA |
| 11 | AF414403 | Hu | GI.3 | Honolulu219 | 1992 | USA |
| 12 | AB187514 | Hu | GI.3 | Otofuke | 1979 | JPN |
| 13 | GQ856470 | Hu | GI.3 | Beijing54108 | 2007 | CHN |
| 14 | JN603244 | Hu | GI.3 | S29 | 2008 | SWE |
| 15 | JN699049 | Hu | GI.3 | C9 | 1978 | GUF |
| 16 | JN699048 | Hu | GI.3 | C91 | 1978 | GUF |
| 17 | JN699050 | Hu | GI.3 | B8 | 1977 | CAF |
| 18 | AB042808 | Hu | GI.4 | Chiba | 1987 | JPN |
| 19 | AJ277621 | Hu | GI.4 | Thistlehall | 1990 | GBR |
| 20 | AF394960 | Hu | GI.4 | Koblenz433 | 2000 | DEU |
| 21 | AJ277614 | Hu | GI.5 | Musgrove | 1989 | GBR |
| 22 | JN699046 | Hu | GI.5 | E57 | 1975 | UGA |
| 23 | AB039774 | Hu | GI.5 | SzUG1 | 2002 | JPN |
| 24 | AF093797 | Hu | GI.6 | BS5 | 1997 | DEU |
| 25 | AF538678 | Hu | GI.6 | VA497 | 1999 | USA |
| 26 | KC998959. | Hu | GI.6 | TCH-099 | 2003 | USA |
| 27 | AY502008 | Hu | GI.6 | Wisconsin | 2001 | USA |
| 28 | AY502007 | Hu | GI.6 | CS-841 | 2001 | USA |
| 29 | AJ277609 | Hu | GI.7 | Winchester | 1994 | GBR |
| 30 | JN005886 | Hu | GI.7 | TCH-060 | 2003 | USA |
| 31 | JN899243 | Hu | GI.7 | Providence191 | 2010 | USA |
| 32 | AJ844469 | Hu | GI.7 | Chiba030100 | 2003 | JPN |
| 33 | AY675555 | Hu | GI.7 | IF2036 | 2003 | IRQ |
| 34 | AF538679 | Hu | GI.8 | Boxer | 2001 | USA |
| 35 | KJ196298 | Hu | GI.8 | Nagoya_KY531 | 2007 | JPN |
| 36 | GU299761 | Hu | GI.8 | 2008890321 | 2008 | USA |
| 37 | GU296356 | Hu | GI.9 | Lilla_Edet | 2008 | SWE |
| 38 | JN183159 | Hu | GI.9 | S48 | 2008 | SWE |
| 39 | HQ637267 | Hu | GI.9 | Vancouver730 | 2004 | CAN |
| 40 | JX289822 | Hu | GII.1 | 7EK-Hawaii | 1971 | USA |
| 41 | AF414416 | Hu | GII.1 | Miami-81 | 1986 | USA |
| 42 | AJ277606 | Hu | GII.1 | Girlington | 1993 | GBR |
| 43 | AF425769 | Hu | GII.1 | Wiesbaden-294 | 2001 | DEU |
| 44 | JN797508 | Hu | GII.1 | Ascension208 | 2010 | USA |
| 45 | JN616378 | Hu | GII.1 | HuN4593 | 2010 | HUN |
| 46 | AB662855 | Hu | GII.2 | OC060083 | 2006 | JPN |
| 47 | AY660568 | Hu | GII.2 | BUDS | 2002 | USA |
| 48 | KC597138 | Hu | GII.2 | CHDC2596 | 1975 | USA |
| 49 | In progress | Hu | GII.2 | Henryton | 1971 | USA |
| 50 | JQ320072 | Hu | GII.2 | NF2002 | 2002 | USA |
| 51 | KC998960 | Hu | GII.2 | TCH560 | 2002 | USA |
| 52 | U22498 | Hu | GII.3 | Mexico | 1989 | MEX |
| 53 | U02030 | Hu | GII.3 | TV24 | 1991 | CAN |
| 54 | KM198573 | Hu | GII.3 | C2H-45 | 2011 | VNM |
| 55 | FJ537134 | Hu | GII.4 | CHDC5191 | 1974 | USA |
| 56 | X76716 | Hu | GII.4 | Bristol | 1993 | GBR |
| 57 | AJ004864 | Hu | GII.4 | Grimsby | 1995 | GBR |
| 58 | AY502023 | Hu | GII.4 | FarmingtonHills | 2002 | USA |
| 59 | DQ078794 | Hu | GII.4 | Hunter284E | 2004 | AUS |
| 60 | EF126965 | Hu | GII.4 | DenHaag89 | 2006 | NLD |
| 61 | EF126963 | Hu | GII.4 | Yerseke38 | 2006 | NLD |
| 62 | AB434770 | Hu | GII.4 | OC07138 | 2007 | JPN |
| 63 | AB445395 | Hu | GII.4 | Apeldoorn317 | 2007 | NLD |
| 64 | GU445325 | Hu | GII.4 | NewOrleans1805 | 2009 | USA |
| 65 | JX459908 | Hu | GII.4 | Sydney_NSW0514 | 2012 | AUS |
| 66 | AJ277607 | Hu | GII.5 | Hillingdon | 1990 | GBR |
| 67 | AF414423 | Hu | GII.5 | WhiteRiver290 | 1994 | USA |
| 68 | AF414422 | Hu | GII.5 | NewOrleans306 | 1994 | USA |
| 69 | AJ277620 | Hu | GII.6 | Seacroft | 1990 | GBR |
| 70 | EF547401 | Hu | GII.6 | Osaka_10203 | 2001 | JPN |
| 71 | AF414408 | Hu | GII.6 | Baltimore_274 | 1993 | USA |
| 72 | KY424341 | Hu | GII.6 | BethesdaD1 | 2012 | USA |
| 73 | GU930737 | Hu | GII.6 | E9913646 | 1997 | USA |
| 74 | AF414410 | Hu | GII.6 | Miami_292 | 1994 | USA |
| 75 | AB682736 | Hu | GII.6 | Ehime090371 | 2009 | JPN |
| 76 | KC464321 | Hu | GII.6 | Ohio_490 | 2012 | USA |
| 77 | AB818397 | Hu | GII.6 | Ehime090646 | 2009 | JPN |
| 78 | AJ277608 | Hu | GII.7 | Leeds | 1990 | GBR |
| 79 | AF414409 | Hu | GII.7 | Gwynedd-273 | 1994 | USA |
| 80 | DQ078846 | Hu | GII.7 | Sydney4477-02S | 2002 | AUS |
| 81 | AF195848 | Hu | GII.8 | Amsterdam-98-18 | 1998 | NLD |
| 82 | AB039780 | Hu | GII.8 | Saitama-U25 | 2002 | JPN |
| 83 | AY038599 | Hu | GII.9 | VA97207 | 1997 | USA |
| 84 | DQ379715 | Hu | GII.9 | G5175 | 1983 | AUS |
| 85 | AY054299 | Hu | GII.9 | Idaho_Falls-378 | 1996 | USA |
| 86 | AF504671 | Hu | GII.10 | Vietnam026 | 2000 | VNM |
| 87 | AY237415 | Hu | GII.10 | Mc37 | 2000 | THA |
| 88 | AF427118 | Hu | GII.10 | Erfurt546 | 2000 | DEU |
| 89 | AB074893 | Sw | GII.11 | Sw918 | 1997 | JPN |
| 90 | AY077644 | Sw | GII.11 | VA34 | 1998 | NLD |
| 91 | HQ392821 | Sw | GII.11 | Ch6 | 2009 | CHN |
| 92 | HM346627 | Sw | GII.11 | DO35 | 2007 | KOR |
| 93 | EU448333 | Sw | GII.18 | F15-10nv | 2007 | CAN |
| 94 | AY823305 | Sw | GII.18 | OH-QW125 | 2003 | USA |
| 95 | AY823304 | Sw | GII.18 | OH-QW101 | 2003 | USA |
| 96 | AY823307 | Sw | GII.19 | OH-QW218 | 2003 | USA |
| 97 | AY823306 | Sw | GII.19 | OH-QW170 | 2003 | USA |
| 98 | AJ277618 | Hu | GII.12 | Wortley | 1990 | GBR |
| 99 | EF547403 | Hu | GII.12 | Akabane2087-990206 | 1999 | JPN |
| 100 | KP064099 | Hu | GII.12 | E5152 | 2010 | FRA |
| 101 | AB078334 | Hu | GII.13 | Kashiwa47 | 2002 | JPN |
| 102 | AY113106 | Hu | GII.13 | Fayetteville | 1998 | USA |
| 103 | EF547405 | Hu | GII.13 | Maizuru000324 | 2000 | JPN |
| 104 | JN699038 | Hu | GII.14 | HK74 | 1978 | CHN |
| 105 | AY130761 | Hu | GII.14 | M7 | 1999 | USA |
| 106 | EF670650 | Hu | GII.14 | Shanxi50106 | 2006 | CHN |
| 107 | AY130762 | Hu | GII.15 | J23 | 1999 | USA |
| 108 | GQ856474 | Hu | GII.15 | Beijing_55161 | 2008 | CHN |
| 109 | KJ196290 | Hu | GII.15 | SapporoHK299 | 2007 | JPN |
| 110 | AY502010 | Hu | GII.16 | Tiffin | 1999 | USA |
| 111 | AY772730 | Hu | GII.16 | Neustrelitz260 | 2000 | DEU |
| 112 | AY502014 | Hu | GII.16 | Fayette | 1999 | USA |
| 113 | KJ196286 | Hu | GII.17 | SaitamaT87 | 2002 | JPN |
| 114 | AY502009 | Hu | GII.17 | CSE1 | 2002 | USA |
| 115 | KC597139 | Hu | GII.17 | C142 | 1978 | GUF |
| 116 | DQ438972 | Hu | GII.17 | Katrina17 | 2005 | USA |
| 117 | GQ266697 | Hu | GII.17 | ZuerichP7d384 | 2009 | CHN |
| 118 | GQ266696 | Hu | GII.17 | Zuerich_P7d1 | 2008 | CHN |
| 119 | KJ156329 | Hu | GII.17 | 13BH1 | 2013 | TWN |
| 120 | KR083017 | Hu | GII.17 | Gaithersburg | 2014 | USA |
| 121 | KP998539 | Hu | GII.17 | CUHK-NS-463 | 2014 | HKN |
| 122 | EU373815 | Hu | GII.20 | Luckenwalde591 | 2002 | DEU |
| 123 | EU424333 | Hu | GII.20 | Leverkusen267 | 2005 | DEU |
| 124 | EU072235 | Hu | GII.20 | CHN42973CZ05 | 2005 | CHN |
| 125 | AB542918 | Hu | GII.20 | OH07011 | 2007 | JPN |
| 126 | AB542917 | Hu | GII.20 | OC07118 | 2007 | JPN |
| 127 | JN899245 | Hu | GII.21 | Salisbury150 | 2011 | USA |
| 128 | KJ196284 | Hu | GII.21 | KawasakiYO284 | 2007 | JPN |
| 129 | AB542915 | Hu | GII.21 | OC05024 | 2005 | JPN |
| 130 | AB083780 | Hu | GII.22 | Yuri | 2003 | JPN |
| 131 | GQ856469 | Hu | GII.22 | Beijing53931 | 2007 | CHN |
