# Supplementary material for: Static and Evolving Norovirus Genotypes: Implications for Epidemiology and Immunity
Source: PLoS Pathog. 2017 Jan 19;13(1):e1006136. doi: 10.1371/journal.ppat.1006136 (PMC5283768; doi:10.1371/journal.ppat.1006136)
Supplement: S4 Table — (DOCX) [file ppat.1006136.s010.docx]

**Table S4.** Sequences of GII.6, GII.12 and GII.17 noroviruses used in this study.

**A. 47 sequences of GII.6 noroviruses (GenBank Accession number and Strain provided)**

| **Accession** | | **Strain** |
| --- | --- | --- |
| 1 | GU930737 | E99-13646 |
| 2 | AF414410 | Miami |
| 3 | AB039778 | SaitamaU16 |
| 4 | AB039779 | SaitamaU17 |
| 5 | AB685741 | OH05010VLP |
| 6 | AB685742 | OC04062 |
| 7 | JX989075 | GZ2010-L96 |
| 8 | JX984953 | GZ2010-L96 |
| 9 | JX984949 | GZ2010-L72 |
| 10 | JX984945 | GZ2010-L1 |
| 11 | AB758451 | Miyagi |
| 12 | AB685740 | OC09015VLP |
| 13 | HM633213 | 8913_Shizuoka |
| 14 | GU969054 | 8915_Maizuru |
| 15 | GU969057 | 9088_Maizuru |
| 16 | GU969056 | 8966_Maizuru |
| 17 | GU969055 | 8920_Maizuru |
| 18 | AB685739 | OH09014 |
| 19 | AB682736 | Ehime090371 |
| 20 | AB685738 | OC08025 |
| 21 | JN183165 | S18 |
| 22 | AJ277620 | Seacroft |
| 23 | EF547401 | Osaka_010203 |
| 24 | AB084071 | GIFU |
| 25 | DQ093064 | 445 |
| 26 | AF414408 | Baltimore_274 |
| 27 | AB039777 | SaitamaU4 |
| 28 | AB039776 | SaitamaU3 |
| 29 | AB078337 | Ueno7k |
| 30 | KC576910 | S9c |
| 31 | JX846927 | CHDC4073 |
| 32 | AF414407 | Florida_269 |
| 33 | KY424341 | BethesdaD1 |
| 34 | JN699041 | HK28 |
| 35 | AB818403 | Ehime031163 |
| 36 | AB818402 | Ehime041525 |
| 37 | AB818401 | Ehime050007 |
| 38 | AB818398 | Ehime090056 |
| 39 | KJ407072 | HS245 |
| 40 | AB818399 | Ehime090549 |
| 41 | KC464321 | Ohio_490 |
| 42 | AB818397 | Ehime090646 |
| 43 | AB685739 | OH09014VLP |
| 44 | AB818400 | Ehime120246 |
| 45 | AB818404 | Ehime030769 |
| 46 | JN699036 | S7e |
| 47 | KY424345 | HenrytonH1 |

**B. 26 sequences of GII.12 noroviruses (GenBank Accession number and Strain provided)**

| **Accession** | | **Strain** |
| --- | --- | --- |
| 1 | HQ449728 | HS210 |
| 2 | GQ845370 | StGeorge-NSW199U |
| 3 | KC464500 | CGMH42 |
| 4 | JQ613568 | Wahroonga-NSW004P |
| 5 | KC464498 | CGMH40 |
| 6 | KC464497 | CGMH39 |
| 7 | HQ401025 | HS207 |
| 8 | HQ664990 | HS206 |
| 9 | JQ613569 | Gunnedah-NSW895P |
| 10 | HQ688986 | Shelby |
| 11 | KC464496 | CGMH38 |
| 12 | HQ115742 | Velence-HUN4417 |
| 13 | AB045603 | Gifu |
| 14 | AB044366 | Hiroshima9912-02F |
| 15 | AF414420 | Honolulu-314 |
| 16 | AB039775 | SaitamaU1 |
| 17 | KF006267 | Texas-E13842 |
| 18 | KP064099 | E5152 |
| 19 | AF397905 | Schwerin003 |
| 20 | EU921353 | Pune-PC24 |
| 21 | AJ277618 | Wortley |
| 22 | EF547403 | Akabane2087-990206 |
| 23 | AB032758 | Aichi76-96 |
| 24 | AF427119 | Pirna-110 |
| 25 | KM198503 | C2033 |
| 26 | KC464499 | CGMH41 |

**C. 144 sequences of GII.17 noroviruses (GenBank Accession number and Strain provided)**

| **Accession** | | **Strain** |
| --- | --- | --- |
| 1 | KC597139 | C142 |
| 2 | DQ438972 | Katrina17 |
| 3 | GQ266697 | ZuerichP7d384 |
| 4 | GQ266696 | Zuerich |
| 5 | JF970609 | INCMNSZ01 |
| 6 | KJ196286 | SaitamaT87 |
| 7 | AY502009 | CSE1 |
| 8 | KT589391 | 2015 |
| 9 | KJ156329 | 13BH1 |
| 10 | LC043168 | Saitama5309 |
| 11 | LC043167 | Saitama5203 |
| 12 | AB983218 | Kawasaki323 |
| 13 | LC043139 | Nagano7-1 |
| 14 | LC043305 | Nagano8-1 |
| 15 | KP902563 | NS-258 |
| 16 | KP902564 | NS-276 |
| 17 | KP902565 | NS-360 |
| 18 | KR083017 | Gaithersburg |
| 19 | KT780402 | NS-549 |
| 20 | KT380915 | 142700_Shanghai |
| 21 | KP998539 | NS-463 |
| 22 | KT780411 | NS-643 |
| 23 | KT780404 | NS-582 |
| 24 | KT780396 | NS-492 |
| 25 | KT780394 | NS-469 |
| 26 | KT780398 | NS-500 |
| 27 | KT780403 | NS-574 |
| 28 | KT780408 | NS-627 |
| 29 | KT780395 | NS-482 |
| 30 | KT780415 | NS-656 |
| 31 | KT780414 | NS-655 |
| 32 | KT780410 | NS-637 |
| 33 | KT780407 | NS-603 |
| 34 | KT780406 | NS-600 |
| 35 | KT780400 | NS-511 |
| 36 | KT780397 | NS-494 |
| 37 | KT780412 | NS-647 |
| 38 | KT780409 | NS-636 |
| 39 | KT780405 | NS-599 |
| 40 | KT780401 | NS-517 |
| 41 | KT780413 | NS-653 |
| 42 | KT780399 | NS-502 |
| 43 | LC037415 | Kawasaki308 |
| 44 | KR154231 | CGMH70 |
| 45 | KT780416 | NS-657 |
| 46 | KR154230 | CGMH69 |
| 47 | KT326180 | NS-405 |
| 48 | KT253245 | ZHITHC-12 |
| 49 | KT970370 | GZ-L313 |
| 50 | KT992790 | HNkaohao_Nanyang |
| 51 | KR020503 | 41621_GZ |
| 52 | KT970369 | GZ-L311 |
| 53 | KT970377 | GZ-L362 |
| 54 | KT970372 | GZ-L325 |
| 55 | KT970376 | GZ-L343 |
| 56 | KT970373 | GZ-L337 |
| 57 | KT970374 | GZ-L339 |
| 58 | KT326182 | NS-667 |
| 59 | KT970371 | GZ-L324 |
| 60 | KT326181 | NS-649 |
| 61 | KT992787 | HN03_Nanyang |
| 62 | KT992786 | HN02_Nanyang |
| 63 | KT992789 | HN05_Nanyang |
| 64 | KT992788 | HN04_Nanyang |
| 65 | KT992785 | HN01_Nanyang |
| 66 | KT970375 | GZ-L340 |
| 67 | KP902590 | NS-575 |
| 68 | KP902589 | NS-574 |
| 69 | KP902588 | NS-565 |
| 70 | KP902587 | NS-556 |
| 71 | KP902580 | NS-512 |
| 72 | KP902567 | NS-438 |
| 73 | KP902569 | NS-456 |
| 74 | KP902568 | NS-455 |
| 75 | KP902570 | NS-463 |
| 76 | KP902571 | NS-480 |
| 77 | KP902572 | NS-482 |
| 78 | KP902573 | NS-483 |
| 79 | KP902574 | NS-492 |
| 80 | KP902575 | NS-493 |
| 81 | KP902576 | NS-500 |
| 82 | KP902577 | NS-502 |
| 83 | KP902578 | NS-503 |
| 84 | KP902579 | NS-506 |
| 85 | KP902581 | NS-514 |
| 86 | KP902582 | NS-517 |
| 87 | KP902583 | NS-520 |
| 88 | KP902584 | NS-521 |
| 89 | KP902585 | NS-528 |
| 90 | KP902586 | NS-549 |
| 91 | KP698931 | NS-513 |
| 92 | KP698930 | NS-511 |
| 93 | KP698929 | NS-494 |
| 94 | KP698928 | NS-491 |
| 95 | KT315719 | NS-671 |
| 96 | KT315718 | NS-670 |
| 97 | KT315716 | NS-662 |
| 98 | KT315715 | NS-659 |
| 99 | KT315714 | NS-658 |
| 100 | KT315713 | NS-657 |
| 101 | KT315712 | NS-656 |
| 102 | KT315711 | NS-655 |
| 103 | KT315710 | NS-653 |
| 104 | KT315709 | NS-650 |
| 105 | KT315707 | NS-648 |
| 106 | KT315706 | NS-647 |
| 107 | KT315705 | NS-643 |
| 108 | KT315704 | NS-641 |
| 109 | KT315703 | NS-639 |
| 110 | KT315702 | NS-637 |
| 111 | KT315701 | NS-636 |
| 112 | KT315700 | NS-634 |
| 113 | KT315699 | NS-629 |
| 114 | KT315698 | NS-627 |
| 115 | KT315697 | NS-619 |
| 116 | KT315696 | NS-616 |
| 117 | KT315695 | NS-612 |
| 118 | KT315694 | NS-611 |
| 119 | KT315693 | NS-606 |
| 120 | KT315692 | NS-604 |
| 121 | KT315691 | NS-603 |
| 122 | KT315690 | NS-602 |
| 123 | KT315689 | NS-600 |
| 124 | KT315688 | NS-599 |
| 125 | KT315687 | NS-593 |
| 126 | KT315686 | NS-592 |
| 127 | KT315685 | NS-589 |
| 128 | KT315684 | NS-586 |
| 129 | KT315683 | NS-582 |
| 130 | KT315682 | NS-579 |
| 131 | KT315681 | NS-570 |
| 132 | KT315680 | NS-560 |
| 133 | KT315679 | NS-539 |
| 134 | KT315678 | NS-537 |
| 135 | KT315677 | NS-534 |
| 136 | KT315676 | NS-533 |
| 137 | KT315675 | NS-523 |
| 138 | KT315674 | NS-522 |
| 139 | KT315673 | NS-486 |
| 140 | KT315672 | NS-478 |
| 141 | KT315671 | NS-476 |
| 142 | KT315670 | NS-475 |
| 143 | KT315669 | NS-472 |
| 144 | KT315668 | NS-469 |
